# Towards a unified theory of plant photosynthesis and hydraulics

**DOI:** 10.1038/s41477-022-01244-5

**Published:** 2022-10-27

**Authors:** Jaideep Joshi, Benjamin D. Stocker, Florian Hofhansl, Shuangxi Zhou, Ulf Dieckmann, Iain Colin Prentice

**Affiliations:** 1 Advancing Systems Analysis Program, International Institute for Applied Systems Analysis, Laxenburg, Austria; 2 Divecha Centre for Climate Change, Indian Institute of Science, Bengaluru, India; 3 Department of Environmental Systems Science, ETH, Universitätsstrasse 2, Zürich, Switzerland; 4 Swiss Federal Institute for Forest, Snow and Landscape Research WSL, Birmensdorf, Switzerland; 5 Biodiversity and Natural Resources Program, International Institute for Applied Systems Analysis, Laxenburg, Austria; 6 Department of Biological Sciences, Macquarie University, Macquarie Park, Australia; 7 CSIRO Agriculture and Food, Glen Osmond, South Australia Australia; 8 Complexity Science and Evolution Unit, Okinawa Institute of Science and Technology Graduate University, Okinawa, Japan; 9 Department of Evolutionary Studies of Biosystems, The Graduate University for Advanced Studies (Sokendai), Hayama, Kanagawa Japan; 10 Department of Life Sciences, Georgina Mace Centre for the Living Planet, Imperial College London, Silwood Park Campus, Ascot, UK; 11 Ministry of Education Key Laboratory for Earth System Modeling, Department of Earth System Science, Tsinghua University, Beijing, China

**Keywords:** Stomata, C3 photosynthesis, Ecophysiology

## Abstract

The global carbon and water cycles are governed by the coupling of CO_2_ and water vapour exchanges through the leaves of terrestrial plants, controlled by plant adaptations to balance carbon gains and hydraulic risks. We introduce a trait-based optimality theory that unifies the treatment of stomatal responses and biochemical acclimation of plants to environments changing on multiple timescales. Tested with experimental data from 18 species, our model successfully predicts the simultaneous decline in carbon assimilation rate, stomatal conductance and photosynthetic capacity during progressive soil drought. It also correctly predicts the dependencies of gas exchange on atmospheric vapour pressure deficit, temperature and CO_2_. Model predictions are also consistent with widely observed empirical patterns, such as the distribution of hydraulic strategies. Our unified theory opens new avenues for reliably modelling the interactive effects of drying soil and rising atmospheric CO_2_ on global photosynthesis and transpiration.

## Abstract (web summary)

Using trait-based optimality theory that unifies stomatal responses and acclimation of plants to changing environments, this study builds a model of the coupling of CO_2_ and water vapour exchanges through the leaves. This successfully predicts the simultaneous decline in carbon assimilation, stomatal conductance and photosynthetic capacity during progressive droughts.

## Main

The fundamental dilemma of plants following the C3 photosynthetic pathway is that when stomata, that is, the tiny ‘valves’ on the surface of leaves, are opened to take in carbon dioxide (CO_2_) for carbon assimilation, water is lost through them via transpiration^1^. The plant’s transpiration stream is maintained by negative water potentials (suction pressures) in roots, transport vessels and leaves. Withstanding negative water potentials requires adapted stem, leaf and root tissues or energy-intensive repair efforts, and extreme water potentials in the xylem can lead to hydraulic failure^2–4^. The risks of hydraulic failure increase when water availability declines across the plants’ rooting zone or when vapour pressure deficit increases at their leaf surfaces. Plants can avoid hydraulic failure by closing their stomatal openings in response to dry soil and atmospheric conditions. However, closing the stomata also leads to a decline in carbon assimilation, creating a tight coupling between carbon uptake and water loss. At the ecosystem level, this coupling of the carbon and water cycles governs the rates of gross primary production (GPP) and evapotranspiration in response to water stress. On one hand, rising atmospheric CO_2_ and increased precipitation are enhancing water use efficiency^5,6^, potentially increasing tree growth rates. On the other hand, rising atmospheric vapour pressure deficits are leading to decreases in stomatal conductance^7^, and rising frequency and intensity of droughts are leading to increased mortality rates^8^. It has been argued that a persistent increase in tree mortality rates, together with a saturating increase in growth rates, is negatively affecting the carbon sink of tropical forests^9^. Accurate predictions of carbon and water fluxes under water stress thus require vegetation models that explicitly account for plant hydraulic processes^10^ to resolve the limiting effect of atmospheric water demand and soil moisture stress on plant photosynthesis^11^.

A plant’s hydraulic machinery places key constraints on how much water it can transpire and, consequently, on its stomatal conductance. Considerable effort has gone into the development of stomatal control models with an explicit treatment of plant hydraulics (see reviews^12,13^). Hydraulically explicit stomatal models have shown success in simulating short-term stomatal responses to drying soil and air on sub-daily and daily timescales^14–17^ and are now being implemented in Earth System Models^18–21^. However, we still lack understanding of how plant physiology acclimates to the development of soil-moisture drought on daily to weekly timescales and how such longer-term acclimation in turn affects stomatal sensitivity to short-term water stress. Such understanding is especially crucial for predicting stomatal and biochemical responses to novel environments and for explaining widely observed patterns related to plant hydraulic strategies (Box 1) in a parsimonious way.

The classic stomatal optimization model^22^ states that plants adjust their stomatal conductance to maximize total carbon assimilation for a fixed amount of water loss, by assuming a constant unit cost for transpired water. This model implies that plants can save water for future use. However, recent stomatal models recognize that plants competitively consume available water^23^. Therefore, an alternative approach conceives the costs of transpiration as arising from the risks of hydraulic failure and the structural and energetic expenditures for withstanding high suction pressures. Thus, many extensions of this classic model explicitly represent plant hydraulics and the associated costs^23–25^. These models require an a priori specification of photosynthetic capacity, which then becomes an additional parameter to be fitted to enable accurate predictions of assimilation rates. By contrast, the least-costs optimization framework^26^ includes the costs of maintaining carboxylation capacity, reflecting a trade-off between investing in photosynthetic and hydraulic capacities^27^. Building upon this approach, a recent model predicts acclimated carboxylation capacity^28^ using the photosynthetic-coordination hypothesis^28,29^. It also explicitly optimizes electron-transport capacity (albeit using a separate optimization criterion)^28^, and has been successful in predicting CO_2_ assimilation rates and leaf-internal CO_2_ concentrations across climatic gradients. However, this model requires an empirical factor to account for the effects of soil moisture^30–32^.

Here we develop a unified first-principles theory combining the photosynthetic-coordination hypothesis with the principles of plant hydraulics within a single optimality framework. Our framework simultaneously predicts the stomatal responses and biochemical acclimation of leaves to environments changing on multiple timescales. We test the resulting model predictions with published data obtained from soil drought experiments conducted with 18 plant species spanning diverse plant functional types. We show that, with just three hydraulic traits and two parameters, our model correctly predicts key observations related to plant photosynthetic responses and hydraulic strategies, as described in Box 1.

Box 1 Widely observed empirical patterns in leaf photosynthetic responses and plant hydraulic strategies as targets for model-based predictions **Stomatal and biochemical responses to soil and atmospheric drought**

1. As soil moisture decreases or vapour pressure deficit (*D*) increases, the first response of leaves is to reduce their stomatal opening to alleviate water stress. Since carbon uptake and water loss occur through stomata, photosynthesis and transpiration both decline with stomatal closure and thus, with decreasing soil moisture^30^.
2. As assimilation declines, maintaining photosynthetic capacity becomes increasingly unprofitable. Therefore, in the short term, leaf photosynthetic capacity also declines with decreasing soil moisture^44,45^. Yet, in the long term, plants acclimate by shedding their leaves, which reduces transpiration demand and allows assimilation to recover. Thus, in the long term, high photosynthetic capacity can be maintained at the expense of a reduced foliage surface area^58^.
3. As *D* increases, the leaf-internal-to-external CO_2_ ratio (*χ*) declines. Various functional forms have been used to describe this decline, these forms having been mostly derived from limited empirical data^77^. A widely used relationship, predicted by simple stomatal optimization models^26,78^, is *χ* = *ξ*/(*ξ* + √*D*), where *D* is expressed as a fraction of atmospheric pressure and *ξ* is a constant. This implies that logit(*χ*) varies linearly with log(*D*) with a slope of −0.5, a value often targeted by modellers^23,28^. However, a recent study^46^ analysing data from hundreds of species along aridity gradients has reported slope values of −0.76 ± 0.15, with remarkable consistency across species. The extent and consistency of these observations suggest that this value ought to be taken as a new target for model-based predictions.

**Hydraulic strategies and trait-adaptations**

4. As soil moisture decreases or *D* increases, xylem water potentials become increasingly negative. Extremely negative water potentials create embolisms in the xylem, which have been linked to increased risks of plant mortality (‘hydraulic failure’). To avoid these risks, plants close their stomata before the onset of substantial xylem embolism^4,37,49,50,79^. At the same time, to maximize carbon assimilation, plants tend to keep their stomata open for as long as possible, often close to the point of hydraulic failure. Thus, plants across species operate at extremely low hydraulic safety margins^52^.
5. Plant traits vary across a continuum of stomatal regulation strategies^67,80^. At one end are isohydric (drought-avoiding) species that maintain a constant leaf water potential by closing the stomata as soil water potential decreases, at the cost of reduced carbon assimilation. At the other end are extreme anisohydric (drought-tolerating) species that keep their stomata open even in the face of decreasing soil water potential to maintain high CO_2_ uptake, at the risk of hydraulic failure. In between are isohydrodynamic species that maintain a relatively constant soil-to-leaf water-potential difference.

## Model summary

We now list the principles and hypotheses underlying our model in general terms, followed by a summary of the optimality framework, plant traits used in our model, the interpretation of model parameters and our strategy for testing the model with experimental data. A detailed model description is presented in Methods, and a full derivation of the model is presented in Supplementary Information section 1.

### Model principles and hypotheses

Our model is based on three principles and hypotheses as follows.

1. Water-balance principle. Any plant must maintain a continuous stream of water across its entire hydraulic pathway (through roots, stems and leaves) to ensure that the atmospheric demand for transpiration is met by the supply of water from the soil^33^. If supply does not equal demand, xylem may embolize or leaves and roots may get damaged, causing catastrophic failure of the hydraulic system. Demand through transpiration depends on the stomatal conductance *g*_s_ and the atmospheric vapour pressure deficit *D*, whereas supply depends on the soil-to-leaf water-potential difference Δ*ψ* and the hydraulic properties of the transpiration pathway. Therefore, this principle predicts *g*_s_ as a function of Δ*ψ* and is widely used in stomatal models that explicitly represent water transport. We use the term ‘principle’ rather than ‘hypothesis’ for this assumption to indicate its rooting in basic physical laws.
2. Photosynthetic-coordination hypothesis. Photosynthetic carbon assimilation is limited by a plant’s capacity for carboxylation *V*_cmax_ and by light availability *I*_abs_, which, together with the electron-transport capacity *J*_max_, determine the rates of biochemical and photochemical reactions governing CO_2_ fixation^34^. In general, the rate of photosynthesis is the minimum of the carboxylation-limited rate *A*_c_ and the light-limited rate *A*_j_. The light-limited rate is further modulated by *J*_max_. Since the carboxylation and electron-transport capacities are costly to maintain, they are hypothesized to acclimate to typical daytime conditions on a weekly timescale, such that the two photosynthetic rates are coordinated, that is, *A*_c_ = *A*_*j*_ ^29,35^.
3. Profit-maximization hypothesis. We posit that, on a weekly timescale (medium-term responses), plants simultaneously optimize their photosynthetic capacity and stomatal conductance to maximize net assimilation (profit, *F*), after accounting for the costs of maintaining photosynthetic capacity and the hydraulic pathway, including the risks of hydraulic failure. On a daily timescale (short-term responses), the acclimated photosynthetic capacities are fixed, and plants can optimize only their stomatal conductance. The parameters scaling the photosynthetic and hydraulic costs, *α* and *γ*, respectively, are the only two latent (that is, not directly observable) parameters in our model and are henceforth called ‘unit costs’.

### Hydraulic pathway and hydraulic traits

**Fig. 1:**
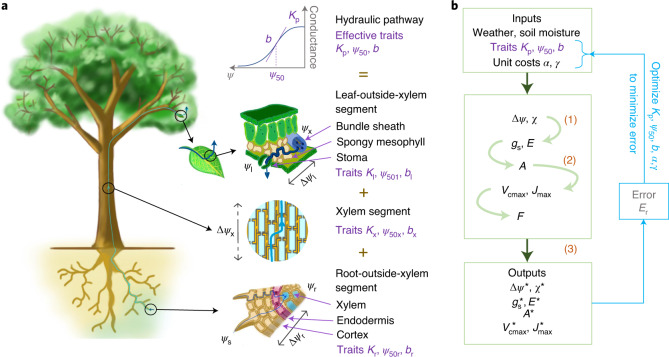
Schematic representation of our model, underlying first principles and notation. **a**, Water-transport pathway. Purple labels indicate the three hydraulic traits that determine the conductance to water flow of each of the three segments of the water-transport pathway. Water potentials are shown at various points along the pathway: *ψ*_s_ in soil, *ψ*_r_ in roots at the beginning of the xylem segment, *ψ*_x_ at the end of the xylem segment and *ψ*_l_ in leaves near the stomata. The soil-to-leaf water potential difference Δ*ψ* = *ψ*_s_ − *ψ*_l_ thus comprises the successive pressure drops along the three segments, that is, Δ*ψ*_r_ = *ψ*_s_ − *ψ*_r_ along the radial outside-xylem segment within the roots, ${\Delta}\psi _\mathrm{x} = \psi _\mathrm{r} - \psi _\mathrm{x}$ along the xylem and ${\Delta}\psi _\mathrm{l} = \psi _\mathrm{x} - \psi _\mathrm{l}$ along the outside-xylem segment within the leaves. **b**, Model-calibration pathway. The model takes as inputs three effective whole-plant hydraulic traits (*K*_p_, *ψ*_50_ and *b*) together with two cost parameters (the unit costs of photosynthetic and hydraulic capacities, *α* and *γ*, respectively). It predicts as outputs the optimal values (denoted by asterisks) of stomatal conductance $g_\mathrm{s}^ \ast$, assimilation rate A^*^, transpiration E^*^, acclimated photosynthetic capacities $V_{{{{\mathrm{cmax}}}}}^ \ast$ and $J_{{{{\mathrm{max}}}}}^ \ast$, soil-to-leaf water-potential difference Δψ^*^ and leaf internal-to-external CO_2_ ratio χ^*^. Each variable is first calculated as a function of Δ*ψ* and *χ*, as shown by the four light-green arrows, from which the optimal combination (Δψ^*^, χ^*^) is then calculated by maximizing profit *F* according to equation (1). Blue arrows and boxes indicate the process through which the best-fit traits and unit costs for each species are calculated by minimizing the model error. Orange labels indicate the three principles and hypotheses underlying the model, displayed next to the processes they affect.

Water from the soil first enters the roots, where it flows through the root cortex, the endodermis and the stele. It then flows through the xylem in roots, the stem and leaf veins. After exiting the xylem, it flows through the bundle sheath and spongy mesophyll cells in the leaf, until it evaporates from the stomatal cell walls and diffuses out to the ambient air^36^ (Fig. 1a). Under moderate water stress, which is the focus of the present work, the outside-xylem segments of the pathway may experience reversible losses in conductivity. For example, the soil-root interface may lose conductivity as roots shrink and disconnect from the soil, while leaves and roots may lose conductivity due to reductions in aquaporin activity or cell membrane permeability^37,38^. Extremely high suction pressures may lead to irreversible loss of xylem conductivity due to cavitation, although some species can reverse it by refilling xylem conduits or growing new xylem^39^. The flow of water through the plant can be described as depending on three effective hydraulic traits, which characterize the combined effect of the individual segments of the pathway: (1) the maximum plant conductance per unit leaf area, that is, the maximum leaf-specific whole-plant conductance *K*_p_, (2) the water potential *ψ*_50_ that causes a 50% loss of whole-plant conductance, and (3) a shape parameter *b* that determines the sensitivity of conductance loss to water potential during progressive drought. There is increasing evidence that roots are the most resistive parts of the hydraulic pathway^40^ and leaves are the most vulnerable^37,41^. This is primarily due to the properties of the outside-xylem segments in roots and leaves, which thus form the hydraulic bottleneck of a plant. Further, there are lags in the recovery of root and stem conductivity upon rehydration, causing hysteresis in the response of the conductivity of these tissues to water potential^42^. While an explicit treatment of such hysteresis may be important for predicting root recovery on short timescales and xylem recovery from extreme drought, we focus on one-way drying in this study, reflecting data limitations and maintaining simplicity and analytical tractability.

### Medium-term responses

To predict the acclimation of photosynthetic capacity on a weekly timescale, we assume that plants independently control their weekly-average stomatal conductance *g*_s_ and their electron-transport capacity *J*_max_ to maximize their net profit *F*, as defined below. After expressing all quantities in Fig. 1b in dependence on *g*_s_ and *J*_max_, or equivalently, in a mathematically more convenient form, in terms of the leaf internal-to-external CO_2_ ratio *χ* and the soil-leaf water-potential difference Δ*ψ* (Methods and Supplementary Information section 1.3), *F* can be written as

1 $$F\left( {\chi ,{\Delta}\psi } \right) = A\left( {\chi ,\,{\Delta}\psi } \right) - \alpha J_{{{{\mathrm{max}}}}}\left( {\chi ,\,{\Delta}\psi } \right) - \gamma {\Delta}\psi ^2,$$

where *A* is the assimilation rate calculated by combining the standard biochemical model of photosynthesis^34^ with the photosynthetic-coordination hypothesis (equation 6 in Methods). We find the optimal solution (χ^*^, Δψ^*^) semi-analytically by first calculating the derivatives of *F* with respect to *χ* and Δ*ψ* analytically (Supplementary equation 16) and then determining their roots numerically.

### Short-term responses

To predict stomatal responses on hourly and daily timescales, we follow a two-step procedure. First, we find the acclimated photosynthetic capacities using the multivariate optimization described above, driven by a 7 d rolling mean of the soil water potential. Once the acclimated *J*_max_ and *V*_cmax_ are known, *A*, *g*_s_ and *χ* can all be expressed in terms of Δ*ψ* alone. We again use the net profit in equation (1) to optimize Δ*ψ*. In this case, we determine *A* as the minimum of the carboxylation-limited rate *A*_c_ and the light-limited rate *A*_j_. Also, since *J*_max_ is fixed, the term *αJ*_max_ becomes constant and can thus be ignored during optimization.

### Interpretation of costs

The photosynthetic costs consist of the costs incurred by maintaining photosynthetic capacities, including the regeneration of RuBP. Since the two photosynthetic capacities are coordinated, these costs are assumed to be proportional to *J*_max_. The hydraulic costs include (1) the construction and respiration costs of stem and leaf tissues, (2) the costs of maintaining osmotic potential and (3) the prospective costs of hydraulic failure. Since these costs are difficult to quantify through mechanistic arguments, we have taken a phenomenological approach and used the expression Δ*ψ*^2^ after assessing various alternative cost expressions (Supplementary Fig. 6). A cost expression that is quadratic in Δ*ψ* has also been adopted previously^23^. Sensitivity of our model predictions to the two cost parameters *α* and *γ* is shown in Supplementary Fig. 5.

### Testing the model with data

We use published data from experiments conducted with 18 species, in which plants were grown in greenhouses under controlled conditions and subjected to progressive soil drought; values of *A* and *g*_s_ (and sometimes also of Δ*ψ*) are reported for different values of predawn leaf water potentials, which are indicative of the soil water potential in the plant’s rooting zone. The dataset has been previously assembled using tables and digitized figures from published literature as detailed in ref. ^43^, which we expanded to include Δ*ψ* measurements. Table 1 lists the sources and the data can be found in Supplementary Datasets 1 and 2. In some experiments, each value of soil water potential was maintained for a long duration, so that photosynthetic capacity could acclimate (species with drought duration =∞ in Table 1). For such species, we use the multivariate optimization model as described above (equation 1). In other experiments, the progression of drought occurred at a natural rate, ranging 12–60 d (Table 1). For such species, we use the two-step procedure outlined above to obtain the instantaneous values of the assimilation rate and stomatal conductance.

**Table 1:** List of species used for testing our model along with their model-estimated trait values

Species	Plant type	Drought duration (days)	Estimated species traits and cost parameters				Original reference
			*K*_p_ (10^−16^ m)	*ψ*_50_ (MPa)	*α*	*γ*	
*Cedrus atlantica*	Gymnosperm	35	0.17	–1.20	0.11	0.10	^68^
*Pseudotzuga menziesii*	Gymnosperm	35	0.20	–1.22	0.10	0.11	^68^
*Glycine max*	Herb	21	2.29	–0.58	0.08	5.00	^69^
*Helianthus annuus*	Herb	12	15.09	–0.35	0.03	5.00	^70^
*Broussonetia papyrifera*	Malacophyll angiosperm	∞	4.53	–0.43	0.10	1.30	^71^
*Platycarya longipes*	Malacophyll angiosperm	∞	1.54	–0.87	0.09	1.30	^71^
*Pteroceltis tatarinowii*	Malacophyll angiosperm	∞	1.86	–0.91	0.11	1.30	^71^
*Allocasuarina luehmannii*	Sclerophyll angiosperm	28	0.75	–1.04	0.12	1.23	^72^
*Cinnamomum bodinieri*	Sclerophyll angiosperm	∞	3.20	–0.61	0.11	1.30	^71^
*Eucalyptus pilularis*	Sclerophyll angiosperm	12	1.71	–0.36	0.10	1.13	^43,73^
*Eucalyptus populnea*	Sclerophyll angiosperm	12	0.94	–1.75	0.10	1.97	^43,73^
*Olea europaea* var. Chemlali	Sclerophyll angiosperm	60	1.95	–0.93	0.07	1.30	^74^
*Olea europaea* var. Meski	Sclerophyll angiosperm	60	1.38	–2.18	0.11	1.30	^74^
*Quercus coccifera*	Sclerophyll angiosperm	12	0.91	–1.08	0.09	0.92	^75^
*Quercus ilex*	Sclerophyll angiosperm	12	1.32	–1.69	0.11	0.70	^75^
*Quercus suber*	Sclerophyll angiosperm	12	2.95	–1.54	0.10	1.86	^75^
*Ficus tikoua*	Shrub	∞	4.31	–0.57	0.12	0.28	^76^
*Rosa cymosa*	Shrub	∞	0.79	–1.38	0.08	0.74	^71^

For each species, data on gas exchange for different values of predawn leaf water potential were obtained from ref. ^43^ that originally compiled them from the sources listed in this table. Three hydraulic traits and two cost parameters were estimated using these data. A value of ∞ for drought duration means that soil water potential was maintained at each value for a long time during the experiment, allowing sufficient time for acclimation. To reduce the degrees of freedom in parameter estimation, we set *b* = 1 for all species, except for *Helianthus annuus* for which *b* was estimated to be 1.4.

## Results

We show that across 18 species, our model correctly predicts photosynthetic responses to the environment. We also show that model-predicted hydraulic strategies for the species in our dataset are consistent with widely observed empirical patterns.

### Photosynthetic responses to soil moisture

**Fig. 2:**
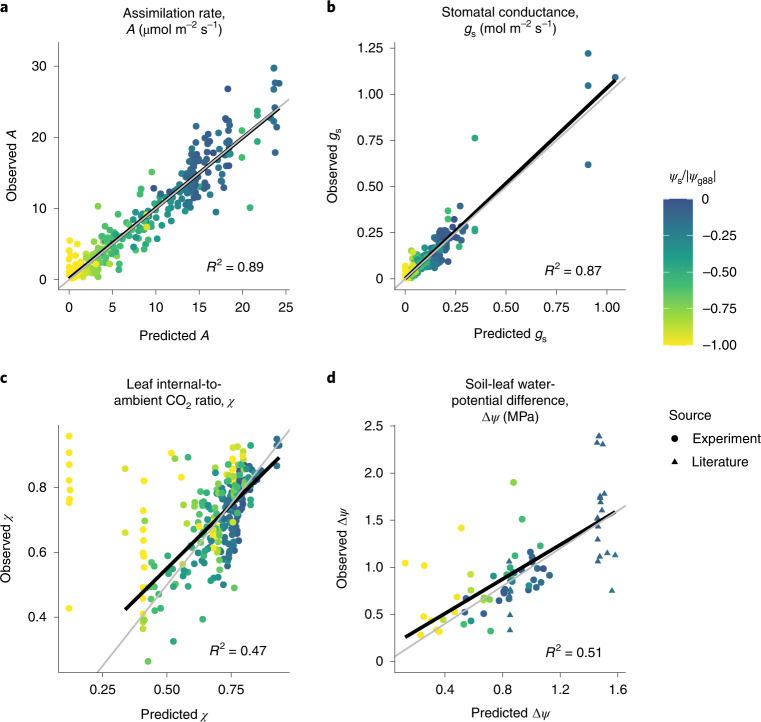
Predicted gas-exchange rates and water relations closely match observations for 18 species. **a**–**c**, Pooled data from all 18 species comparing assimilation rate *A* (**a**), stomatal conductance *g*_s_ (**b**) and leaf-internal-to-external CO_2_ ratio *χ* (**c**) for different values of soil (predawn leaf) water potential *ψ*_s_. **d**, Predicted values of soil-to-leaf water-potential difference Δ*ψ* compared to observations for (1) six species for which midday leaf water potentials were reported in the corresponding experiments, and thus measured under the same environmental conditions as the gas-exchange rates (circles), and (2) two species (*Pseudotzuga menziesii* and *Olea europea* var. Meski) for which values were obtained from literature ^67^ (triangles). Colours indicate soil water potential relative to the stomatal closure point (*ψ*_g88_) of the species; thus, yellow points represent soil water potentials at or beyond stomatal closure. Black lines show linear regressions, while grey lines are the 1:1 lines that represent perfect predictions. In **c**, we ignore points with *ψ*_s_ < *ψ*_g88_ (yellow points) while calculating the regression line, since there is a known bias in predictions of *χ* beyond stomatal closure (see Discussion).

**Fig. 3:**
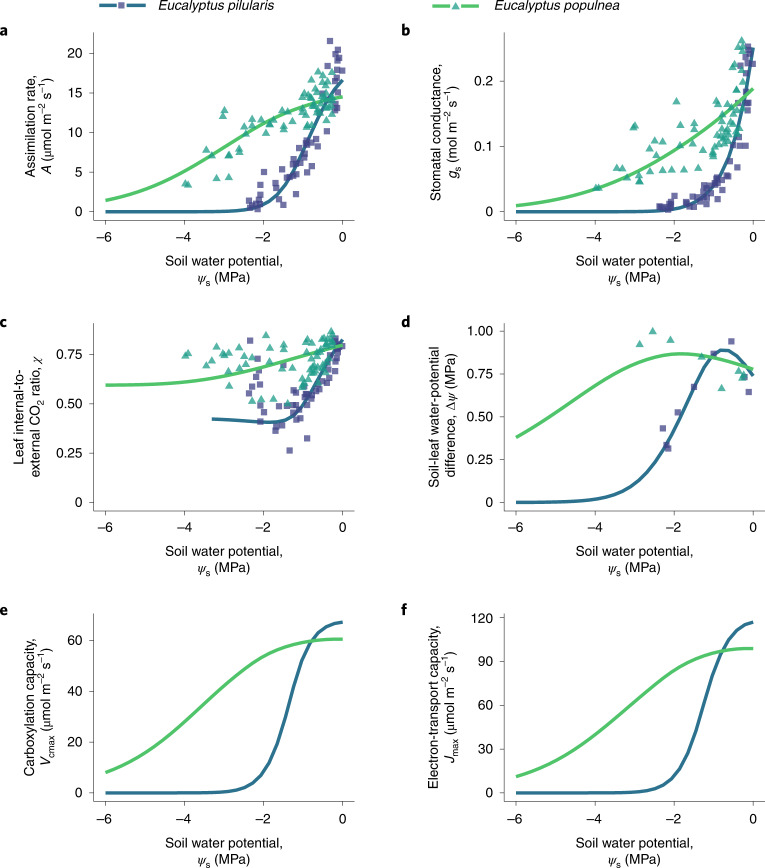
Predicted photosynthetic responses to progressive soil drought closely match observations. Matches are shown here for two *Eucalyptus* species from contrasting climates, and corresponding matches for all 18 species are shown in Supplementary Fig. 1. **a**–**f**, Predicted responses (lines) and observed responses (points) to decreasing soil water potential (*ψ*_*s*_, measured as predawn leaf water potential): assimilation rate *A* (**a**), stomatal conductance *g*_*s*_ (**b**), leaf-internal-to-external CO_2_ ratio *χ* (**c**), soil-to-leaf water-potential difference Δ*ψ* (**d**), carboxylation capacity *V*_cmax_ (**e**) and electron-transport capacity *J*_max_ (**f**). *Eucalyptus pilularis* (blue lines and squares) typically occupies warm and humid coastal areas in eastern Australia, whereas *Eucalyptus populnea* (green lines and triangles) typically occupies semi-arid interior regions of eastern Australia. Since both species were grown in the same greenhouse during the experiment, their contrasting responses reveal genetic adaptations to their native environments. For both species, progressive drought was experimentally induced over 12 d, resulting in a fast instantaneous response of stomatal conductance in combination with a slow acclimating response of photosynthetic capacity. Our model predictions readily account for both responses.

Our model correctly predicts the variation in assimilation rate (*A*), stomatal conductance (*g*_s_), leaf-internal-to-external CO_2_ ratio (*c*_i_:*c*_a_, or *χ*) and soil-to-leaf water-potential difference (Δ*ψ*) in response to soil-moisture availability (*ψ*_s_; Fig. 2). Specifically, the shapes of these dependencies closely resemble those observed during experimental drought: Fig. 3 shows predicted and observed responses for two *Eucalyptus* species from contrasting habitats, and Supplementary Fig. 1 shows the corresponding responses for all 18 species. Moreover, cross-validation analysis shows that our model generalizes to out-of-sample soil-moisture conditions (Supplementary Table 1).

Empirical studies report that photosynthetic capacity (*V*_cmx_ and *J*_max_) declines in response to developing soil drought^44,45^. A unique feature of our model is its ability to predict these responses correctly, qualitatively in accordance with these studies (Fig. 3e,f). Since *χ* depends on both *J*_max_ and *g*_s_, correct predictions of *χ* require predicting both quantities correctly. Therefore, a close match between predicted and observed values of *χ* (Fig. 2c) provides further quantitative validation of the photosynthetic capacity predicted by our model.

### Photosynthetic responses to vapour pressure deficit

Our work builds on the principles introduced by ref. ^28^, and thus inherits the capacity to accurately predict^32^ photosynthetic responses to temperature, atmospheric CO_2_ and light intensity (Supplementary Fig. 2c–e). Furthermore, by explicitly accounting for plant hydraulics, our model delivers improved predictions of photosynthetic responses to soil moisture and vapour pressure deficit (Supplementary Fig. 2a,b).

**Fig. 4:**
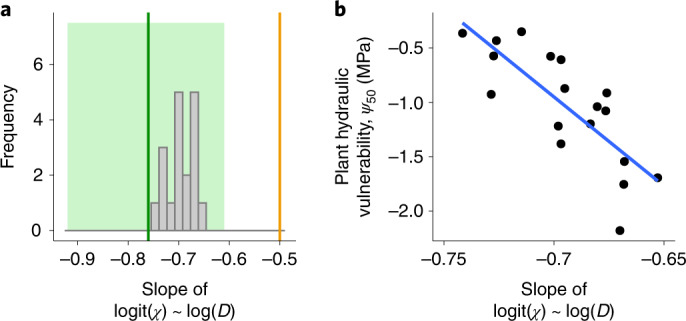
Our model correctly predicts the response of *χ* to vapour pressure deficit. **a**, The model-predicted distribution of the slope of the relationship between logit(*χ*) and log(*D*) for the analysed species (grey bars) is well within the range reported in ref. ^46^ (their reported mean and confidence interval is shown by the green line and green region, respectively). It is significantly different from −0.5 (orange line; a one-sample *t*-test shows a predicted mean slope of −0.7 and a 95% confidence interval of (−0.72, −0.68)). For each species, we calculate the predicted slope by varying vapour pressure deficit in the range 5–5,000 Pa while keeping other environmental parameters constant (at values reported in the respective experiments, with *ψ*_s_ = 0) and using fitted trait values (Table 1). **b**, This slope is correlated with the *ψ*_50_ (black points are species-specific values and the blue line is a linear regression line), with more negative slopes observed for species with less negative *ψ*_50_ (drought avoiders). This could be a reason why earlier datasets supported a slope value of −0.5, as such datasets were often dominated by temperate evergreen species, which are typically characterized by highly negative values of *ψ*_50_.

The functional relationship between logit(*χ*) and log(*D*) predicted by our model shows a close match with observations (Box 1, point 3). In particular, our model predicts this relationship to be linear, with a median slope value of −0.697 and 5%–95% quantile range of (−0.75, −0.67) (Fig. 4a). These predicted slope values are well within the confidence interval reported in the literature^46^. Also, we find that this slope is negatively correlated with *ψ*_50_ (Fig. 4b), such that species with highly negative *ψ*_50_ have less negative slope values. Since earlier datasets were dominated by temperate evergreen species, this could explain why a slope value of −0.5 predicted by previous models was supported by such datasets. We offer our predicted correlation between the slope and *ψ*_50_ as an empirically testable prediction for future studies.

### Predicted hydraulic strategies match observations

In this section, we compare several widely observed empirical patterns among plant hydraulic traits with the corresponding model-predicted patterns. This qualitative comparison allows us to validate our model at an even deeper level.

**Fig. 5:**
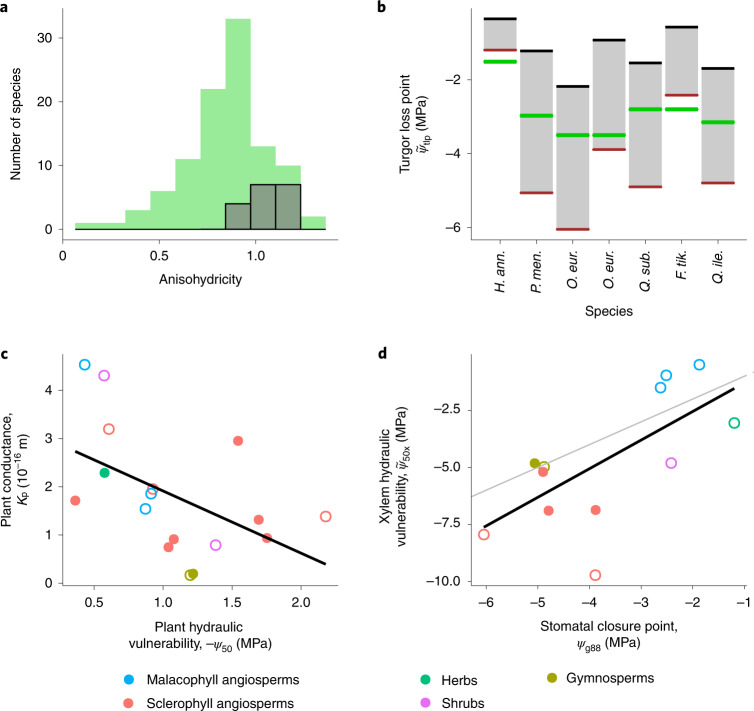
Our model predictions are consistent with widely observed empirical patterns. **a**, The predicted distribution of the degree of anisohydricity among the 18 analysed species (grey bars) lies within the observed global distribution (green bars; as reported in ref. ^47^). **b**, Consistent with empirical observations, the observed turgor loss point (thick green line) lies between the model-predicted water potential at 50% loss of plant conductivity (*ψ*_50_; black line) and the model-predicted water potential at 88% stomatal closure (*ψ*_g88_; brown line). **c**, Plant hydraulic conductance (*K*_p_) is weakly negatively correlated with *ψ*_50_, with no species having high values of both traits, implying a weak safety-efficiency trade-off in line with empirical observations. **d**, When leaf water potential is at *ψ*_g88_, the observed loss of xylem conductivity is typically less than 50% (implied by observed xylem hydraulic vulnerability $\tilde \psi _{50\mathrm{x}}$ being less than model-predicted *ψ*_g88_), which means that plants close their stomata before the onset of substantial xylem embolism. Furthermore, the difference between the regression line (black) and the 1:1 line (grey) is low, implying that the hydraulic safety margin $\tilde \psi _{50\mathrm{x}} - \psi _{\mathrm{g}88}$ is small on average. Sources for values of $\tilde \psi _{50\mathrm{x}}$ are given in Supplementary Table 2. Closed circles indicate species for which *γ* was estimated using data on Δ*ψ*, whereas open circles refer to species for which such data were not available and for which we therefore used an average value of *γ* estimated for the respective plant types.

First, we compare the distribution of the model-predicted degree of anisohydricity for the 18 analysed species (Box 1) to an empirically observed distribution obtained from a recently compiled database on 102 species across the globe^47^. For each species, the degree of anisohydricity is determined by the slope of the relationship between the water potential in the leaf (*ψ*_l_) and in the soil (*ψ*_s_), measured at low *ψ*_s_ (slope <1 for isohydric, =1 for isohydrodynamic and >1 for anisohydric species). The observed global distribution of these slopes peaks at approximately 1, suggesting that the global majority of species follow the isohydrodynamic strategy^47^. The corresponding distribution predicted by our model lies within the observed distribution (Fig. 5a). Similarly, the predicted distribution of typical operating water potentials (*ψ*_l_ at *ψ*_s_ = 0) also closely matches the corresponding empirically observed distribution (Supplementary Fig. 3).

Second, we compare the relationship between model-predicted plant hydraulic vulnerability and empirically observed turgor loss point for a subset comprising 7 of the 18 analysed species for which these empirical data were available. Empirical observations show that the turgor loss point lies between the point where the leaf loses 50% conductivity (*ψ*_50_) and the point of stomatal closure (*ψ*_g88_)^48^. Our model predictions are consistent with this observation for most of those 7 species (Fig. 5b).

Third, we compare how model-estimated plant hydraulic vulnerability (*ψ*_50_), a measure of hydraulic safety, covaries with model-estimated plant hydraulic conductance (*K*_p_), a measure of hydraulic efficiency. Global data reveal a trade-off between safety and efficiency, that is, no plants score high on both, but only a weak correlation between them, that is, many plants score low on both. Consistent with these observations, we find only a weak correlation between model-estimated values of *ψ*_50_ and *K*_p_, with a few species having low values of both traits, but no species having high values of both (Fig. 5c).

Fourth, we compare how the model-estimated stomatal closure point (*ψ*_g88_) relates to empirically observed xylem hydraulic vulnerability ($\tilde \psi _{50\mathrm{x}}$) for the 18 analysed species. Empirical observations show that stomatal closure occurs before the onset of substantial xylem embolism^37,49–51^, which is probably an adaptation to prevent plant mortality during drought^4^. At the same time, the minimum water potential experienced by the leaves (*ψ*_min_) is close to the water potential at which the xylem loses 50% conductivity ($\tilde \psi _{50\mathrm{x}}$), leading to extremely low hydraulic safety margins^52^. Both of these observations are matched by our model, confirmed by a close correlation between *ψ*_g88_ (which is a proxy of *ψ*_min_) and $\tilde \psi _{50\mathrm{x}}$ (Fig. 5d).

## Discussion

We have presented an analytical trait-based optimality model, unifying plant photosynthesis and hydraulics to predict the stomatal responses and biochemical acclimation of plants to changing hydroclimates. Consistent with widely observed empirical patterns and benchmarked with experimental data available for 18 different plant species, our model correctly predicts the stomatal and photosynthetic responses to soil drought and the dependencies of photosynthesis on vapour pressure deficit, temperature, light intensity and CO_2_.

### Comparison with other stomatal optimization models

To our knowledge, our model is probably the first to combine semi-analytical simplicity with physiological realism to predict the simultaneous stomatal and biochemical responses of plants to the environment, including water stress. Our approach has four key strengths: (1) the multivariate optimization used in our model allows for predicting the observed s-shaped decline of *V*_cmax_ in response to drying soil from first principles, in contrast to most other models that require specification of *V*_cmax_ as a species-specific trait; (2) explicit inclusion of plant hydraulics in our model enables separating the stomatal responses to atmospheric drought and soil-moisture availability, which could be used for improving remote-sensing estimates of GPP; (3) being based on optimality principles and specified with just two latent parameters, our model can be expected to perform well even under out-of-sample environmental conditions, that is, those outside the domain of environmental factors used for calibration, such as elevated CO_2_ levels; and (4) enabling a semi-analytical solution, our model is computationally efficient and can thus be readily incorporated into existing vegetation modelling frameworks. A quantitative model intercomparison between stomatal models including ours would provide valuable insights into the relative accuracy and strengths of different stomatal optimization frameworks and would thus be an interesting direction for future research.

Leaf photosynthesis is known to be jointly constrained by stomatal and non-stomatal limitations. A vast majority of photosynthesis models account only for stomatal limitations, where stomatal conductance is optimized to maximize photosynthetic gain. Non-stomatal limitations, such as the constraints imposed by leaf mesophyll, photosynthetic capacity and sugar transport, have received attention only in the most recent stomatal models. Even such models account for them using a pre-determined functional response, in which mesophyll conductance^53^ or photosynthetic capacity^24,53,54^ is scaled in a prescribed way with stomatal conductance. By contrast, our model can account for non-stomatal limitations without the need to specify photosynthetic capacity a priori. This is especially important when applying it to out-of-sample environmental conditions and to species for which empirical estimates of photosynthetic capacity are not available.

Almost all current models of stomatal optimization focus on water transport through the xylem. By contrast and in line with growing evidence^37,40–42^, we hypothesized that the outside-xylem segments of the hydraulic pathway (in the leaves and roots) together form the hydraulic bottleneck of the plant. The hydraulic-segmentation hypothesis^55^ states that expendable organs such as leaves and fine roots act as a ‘safety valve’ by losing conductivity and driving stomatal closure before the onset of fatal xylem cavitation. Therefore, we hypothesized that our model-based estimates of *ψ*_50_ would arise from leaves and roots and thus be less negative than the corresponding empirically observed values for xylem ($\tilde \psi _{50\mathrm{x}}$). Our findings are consistent with these hypotheses. We offer the fitted values of these traits (*ψ*_50_ and *K*_p_) as testable predictions that can be validated by explicitly measuring these traits for the leaves and roots of the corresponding species.

### Model assumptions and extensions

Under extreme hydroclimatic conditions, such as extremely dry or flooded soils, or extremely low atmospheric CO_2_ levels, our model predictions may deviate slightly from observations. For example, most species in our dataset show an increase in the leaf-internal-to-external CO_2_ ratio (*χ*) after stomatal closure (Fig. 3). The model-predicted *χ* does not increase, asymptotically approaching a constant value instead (Fig. 3 and Supplementary equation 18). The increase in *χ* is due to a build-up of CO_2_ in the leaf, which can happen via two mechanisms: (1) if dark respiration continues even after assimilation has ceased, or (2) if assimilation and respiration decline together due to reduced photosynthetic activity while CO_2_ continues to ‘leak in’ through the leaf cuticle^56^. Future research could identify which of these processes is observed in leaves: since the source of CO_2_ is different in the two mechanisms (plant metabolism or ambient air), they can be distinguished on the basis of whether the build-up is detected also in δ^13^C measurements. Our model could be extended to include these mechanisms, but this would not affect the predicted assimilation rates because these mechanisms are relevant only after stomatal closure.

For simplicity, we have assumed a single effective hydraulic pathway characterized by effective traits summarily describing water flow through the different segments (leaves, xylem and roots) of the pathway. However, a more realistic extension of our model could readily be developed by explicitly modelling water transport through each segment. In Supplementary Information section 2, we present a derivation of an extended model accounting for a multi-segment pathway. Such an extension would be particularly useful for resolving the roles of roots and leaves in stomatal control and drought survival.

To avoid making the model too complex and parameter-heavy and to enable a semi-analytical solution, we have neglected two physiological details, inclusion of which offers promising directions for further research: (1) the leaf’s energy balance and (2) hysteresis in the conductivity-soil-moisture relationship. First, when soil dries, reduced transpiration raises the leaf temperature, which in turn affects the temperature-dependent photosynthetic parameters and the dark-respiration rate. Second, the recovery of root and stem conductivity after rehydration is typically slower than the corresponding loss of conductivity during dehydration^42^. This leads to hysteresis in the relationship between conductivity and soil water potential. The speed of recovery is especially hampered under extreme drought when xylem becomes cavitated because recovery from cavitation requires embolism refilling or growth of new xylem. In our model, the relationship between soil water potential and conductivity is described by a vulnerability curve *P*(*ψ*) that does not include hysteresis. To account for hysteresis, one may use a separate vulnerability curve during recovery or use a hysteretic submodel for conductivity^39^. Further research focusing on drought and rewetting experiments can help generate data for robustly parameterizing and validating such a model.

Plants respond to increasing water stress on multiple timescales and at multiple scales of organization (leaves, whole plants and even stands). Our model accounts for leaf-level responses on daily and weekly timescales, capturing the role of stomatal closure in preventing damagingly negative stem water potentials. Scaling responses from the leaf level to the whole-plant level requires considering the distribution of leaves within the plant canopy as well as the total canopy area: this could be achieved by embedding our model within a model of plant canopies. Such a model could then be extended further to yield optimality-based first-principles models for the plant level^57^, accounting for responses beyond the point of stomatal closure, such as the shedding of leaves on a monthly timescale, which occurs either in full to prevent loss of water through cuticular tissue^4^ or in part to reduce transpiration demand and continue photosynthesis^58^. Similarly, changes to the characteristics and architecture of the transpiration pathway, as reflected in stem traits^59^, could be modelled on yearly to decadal timescales. Trait adaptation on centennial or millennial timescales could be modelled by embedding our leaf-level optimality theory into evolutionary models^60–62^.

In our model, on the shortest timescale (minutes to days), plants may optimize leaf water potential for a fixed (acclimated) photosynthetic capacity. On the timescale of weeks, plants additionally adjust their photosynthetic capacities. In principle, the weekly timescale can be modelled either with nested optimization (that is, optimizing daily, or sub-daily, stomatal conductance for a given photosynthetic capacity, and optimizing weekly photosynthetic capacity by maximizing the total profit over a week), or with simultaneous optimization (that is, optimizing both variables together by assuming a constant environment during the week, representative for mean daytime conditions). In this work, we have taken the latter approach for theoretical and computational simplicity, but the alternative nested approach is worth exploring in future work.

### Implications for global vegetation modelling

Taking advantage of the increasing availability of global data on plant traits, our model can be applied at the global scale by making a few additional assumptions. For the species in our dataset, we find that the photosynthetic unit cost *α* lies within a narrow range of values (0.08–0.12), which could therefore be treated as a constant. Furthermore, variations in the hydraulic unit cost *γ* are primarily driven by differences between plant types, with relatively lesser variability within plant types, suggesting that *γ* could also be treated as a constant within plant types (Supplementary Fig. 4). To infer the global distribution of *α* and *γ*, our model can be used in a Bayesian framework on global data on gas-exchange measurements. When plant-level hydraulic traits *ψ*_50_ and *K*_p_ are not available, these could be derived from other widely measured traits on the basis of observed patterns of functional coordination among plant organs. As a starting point, we have shown that *K*_p_ is weakly correlated with *ψ*_50_ (Fig. 5c) and specific leaf area (Supplementary Fig. 4b). Further studies could test for similar correlations with leaf vein density and root mass fraction, which are respectively expected to affect leaf and root conductivities.

Accurate models of plant photosynthesis are crucial for improving projections of the global carbon and water cycles, because photosynthesis and transpiration by terrestrial plants account for 56% and 30% of the global fluxes of carbon dioxide and water, respectively^63,64^. It is especially important to develop models that can generalize to new climatic conditions, because the projected increase in the frequency and intensity of droughts may lead to unprecedented climatic conditions in future. The inclusion of plant hydraulics into vegetation models has been shown to improve predictions of global productivity and evapotranspiration^17–21^, as well as predictions of the spatiotemporal diversity of vegetation^65^. Spearheading the development initiated by these studies, our model is ideally suited for being embedded into global vegetation models: by accounting for biochemical acclimation, plant hydraulics and photosynthetic trade-offs through optimality principles, our model can extend to new species and new environmental conditions with a raised degree of confidence. Furthermore, accounting for photosynthetic and hydraulic costs is bound to yield more accurate estimates of the energy spent on resource acquisition and, consequently, of the resources available for growth and reproduction. Therefore, embedding our model of photosynthesis and hydraulics into a demographic model of plant communities can help improve the scaling of assimilation and transpiration from the leaf level to the whole-plant level, and even from plants to communities, thus paving the way for more accurate and robust land-surface models.

## Methods

Our model consists of three key components or modules, corresponding to the three principles and hypotheses: a water-transport module to account for plant hydraulics and water balance, a photosynthesis module to account for photosynthesis and the photosynthetic-coordination hypothesis, and a profit-maximization module to implement the optimization. Here we describe the equations used for each module, as well as our strategy for model calibration. Full derivations of the equations can be found in Supplementary Information section 1.

### Water-transport module

We model water transport using Darcy’s law applied to small cross-sections of the hydraulic pathway (Supplementary Information section 1.1.4). In principle, our model of water transport can explicitly represent multiple segments (Supplementary Information section 2), but for simplicity, we represent the entire pathway by a single ‘effective segment’ with traits *K*_p_, *ψ*_50_ and *b*. Thus, our hydraulic model is mathematically similar to the model for xylem water transport described in ref. ^24^, but the effective hydraulic traits in our model correspond not necessarily to the xylem but to the whole plant. As the outside-xylem segments (in leaves and roots) are the hydraulic bottlenecks of the plant for many species^37,40–42^, the traits modelled here probably correspond to these segments.

The conductivity *κ* of any cross-section of the pathway declines as the water potential becomes increasingly negative. This decline in conductivity is phenomenologically described by a so-called vulnerability curve *P*(*ψ*), such that *κ*(*ψ*) = *κ*(0)*P*(*ψ*). The vulnerability curve is typically described by a Weibull function with two parameters: the water potential *ψ*_50_ at which 50% conductivity is lost and a shape parameter *b* that determines the sensitivity of conductivity loss to water potential,

2 $$P\left( \psi \right) = \left( {1/2} \right)^{\left( {\psi /\psi _{50}} \right)^b}.$$

Water potential drops continuously along the hydraulic pathway, from *ψ*_s_ in the soil to *ψ*_l_ at the leaf surface, with a continuous decline in conductivity along the pathway. The volumetric flow of water per unit leaf area in the pathway, *Q*, is therefore described by a differential equation (see Supplementary Information section 1.1.4 for derivation), which can be solved for *Q* as follows,

3 $$Q = - \frac{{\kappa _\mathrm{p}}}{{L\eta }}{\int}_{\psi _\mathrm{s}}^{\psi _\mathrm{l}} {P\left( \psi \right)d\psi ,}$$

where κ_p_ is the effective conductivity of the whole plant per unit leaf area, *L* is the effective length of the hydraulic pathway and *η* is the dynamic viscosity of water. The composite term κ_p_/*L* = *K*_p_ is the whole-plant conductance per unit leaf area (Supplementary Information section 1.1). To keep the number of parameters low to prevent overfitting of the model to data, we use equation (3) to model water flow. In Supplementary Information section 2, we propose an extension of this model, deriving an expression for *Q* by explicitly accounting for segments of the hydraulic pathway in the roots, stem and leaves.

The water-balance principle states that the atmospheric demand for water imposed by vapour pressure deficit at the leaf surface matches the supply of water from the soil via the stem and leaf segments of the hydraulic pathway. The transpiration rate at which water vapour diffuses out of the leaf into the atmosphere is given by (see Supplementary Information section 1.1.6 for derivation)

4 $$E = 1.6g_\mathrm{s}D,$$

where *g*_s_ is the stomatal conductance and *D* is the atmospheric vapour pressure deficit divided by the atmospheric pressure. This rate *E* equals the rate *Q* at which water enters the leaf according to equation (3), which allows us to calculate *g*_s_ by solving

5 $$- \frac{{K_\mathrm{p}}}{\eta }{\int}_{\psi _{{{\mathrm{s}}}}}^{\psi _{{{\mathrm{s}}}} - {\Delta}\psi } {P\left( \psi \right){{{\mathrm{d}}}}\psi = 1.6g_{{{\mathrm{s}}}}D.}$$

### Photosynthesis module

The assimilation rate *A* is calculated from the Farquhar-von Caemmerer-Berry biochemical model^34^ (Supplementary Information section 1.2.1), with the photosynthetic capacities *J*_max_ and *V*_cmax_ linked through the photosynthetic-coordination hypothesis (Fig. 1b and Supplementary Information section 1.2.4). Temperature responses of photosynthesis parameters, such as the Michaelis-Menten coefficient and the light-compensation point, are modelled with Arrhenius functions for enzymatic rates as previously described^31^.

The photosynthetic-coordination hypothesis states that under typical daytime conditions, assimilation operates at the point of co-limitation, such that the carboxylation-limited and light-limited assimilation rates are equal. With this assumption, the co-limited assimilation rate can be written as (Supplementary Information section 1.2)

6 $$A = \frac{J}{4} \cdot \frac{{\chi c_{{{\mathrm{a}}}}(1 - b_{{{\mathrm{r}}}}) - ({\Gamma}^ \ast + b_{{{\mathrm{r}}}}K_{{{\mathrm{M}}}})}}{{\chi c_{{{\mathrm{a}}}} + 2{\Gamma}^ \ast }},$$

where *J* is the effective electron-transport capacity, which increases with light availability *I*_abs_ but saturates due to limitation by the leaf’s intrinsic maximum electron-transport capacity *J*_max_,

7 $$J = \frac{{4\phi _0I_{{{{\mathrm{abs}}}}}}}{{\sqrt {1 + \left( {\frac{{4\phi _0I_{{{{\mathrm{abs}}}}}}}{{J_{{{{\mathrm{max}}}}}}}} \right)^2} }}.$$

Here, *c*_a_ is the atmospheric CO_2_ concentration, *χ* is the ratio of the leaf-internal and external CO_2_ concentrations (*c*_i_:*c*_a_), ${\Gamma}^ \ast$ is the light-compensation point, *K*_M_ is the Michaelis-Menten coefficient for C3 photosynthesis, *ϕ*_0_ is the quantum yield efficiency, *I*_abs_ is the absorbed photosynthetically active radiation and *b*_r_ is the ratio of dark respiration to carboxylation capacity (dark respiration is assumed to be proportional to carboxylation capacity, that is, *R*_d_ = *b*_r_*V*_cmax_). Temperature dependencies of ${\Gamma}^ \ast$ and *K*_M_ are modelled according to ref. ^31^. The ratio *b*_r_ also has a weak dependence on temperature^66^, which we have ignored in this work. Variation in *J*_max_ in response to light and water availability (by optimization) implies a coordinated variation in both carboxylation and electron-transport capacities.

### Profit-maximization module

We assume that plants maximize net assimilation (or profit, *F*) defined in equation (1). Without loss of generality, we also assume that the unit benefit of assimilation is one, that is, *α* and *γ* represent the ratios of the unit costs to unit benefits of assimilation. To optimize equation (1), we express all quantities in terms of the two independent variables *χ* and Δ*ψ* and set the gradient of the profit function to 0. This can be done analytically (Supplementary equation 16). However, except in the special case of strong *J*_max_ limitation, the roots of the gradient must be found semi-analytically (Supplementary Information section 1.3.2). Solving for optimal χ^*^ and Δψ^*^ in turn allows us to predict the optimal photosynthetic capacities ($V_{{{{\mathrm{cmax}}}}}^ \ast$ and $J_{{{{\mathrm{max}}}}}^ \ast$), stomatal conductance ($g_{{{\mathrm{s}}}}^ \ast$) and CO_2_ assimilation rate (A^*^).

### Parameter estimation and model testing

We drive the model with environmental variables (temperature, vapour pressure deficit, light intensity and CO_2_) as specified in the experimental studies. Other parameters used in the model are as follows: *ϕ*_0_ = 0.087, *b*_r_ = 0.002. In the case of instantaneous responses, we use the daily maximum light intensity under growth conditions to calculate the acclimated response, and saturating light intensity (as specified in the studies) to calculate the instantaneous response, so as to mimic the conditions present during LiCor measurements.

Since measurements of effective whole-plant hydraulic traits are not readily available, we treat them as model parameters and estimate them along with the two cost parameters. Values of Δ*ψ* were reported for 6 of the 18 species from the same drought experiments. We supplement Δ*ψ* observations with typical values reported in the literature^67^ for two additional species (*Pseudotsuga menziesii* and *Olea europea* var. Meski). For such species (for which measurements of Δ*ψ* are available), we calibrate five parameters (*α*, *γ*, *ψ*_50_, *b*, *K*_*p*_) by minimizing the sum of squared errors (*E*_r_) between predicted and observed values of *A*, *g*_s_, *χ* and Δ*ψ*, defined as

8 $$E_\mathrm{r} = \mathop {\sum}\limits_i {\left( {\frac{{A_i - \tilde A_i}}{{E[\tilde A_i]}}} \right)^2 + \mathop {\sum}\limits_i {\left( {\frac{{g_{\mathrm{s},i} - \tilde g_{\mathrm{s},i}}}{{E[\tilde g_{\mathrm{s},i}]}}} \right)^2 + \mathop {\sum}\limits_i {\left( {\frac{{\chi _i - \tilde \chi _i}}{{E[\tilde \chi _i]}}} \right)^2 + \mathop {\sum}\limits_i {w_i\left( {\frac{{{\Delta}\psi _i - \widetilde {{\Delta}\psi _i}}}{{E[{\Delta}\psi _i]}}} \right)^2,} } } }$$

where *i* represents different values of *ψ*_s_, *E*[] denotes the mean value, and variables with tilde (for example, $\tilde \chi$) represent observations.

From this calibration, we obtain the mean estimated value of *γ* for each plant type. For all other species (for which measurements of Δ*ψ* are not available), we use this mean value of *γ* and estimate the remaining four parameters. To further reduce the degrees of freedom in model parameterization, we fix the value of *b* = 1 for all species, except for *H. annuus* for which *b* also had to be estimated. For each species for which data on Δ*ψ* are available, we evaluate model performance using fivefold cross-validation (or leave-one-out cross-validation when data points are limited).

### P-hydro R and C++ packages

R code to run our model (P-hydro) is provided as an extension of the ‘rpmodel’ package (the version used in this paper is archived at https://github.com/jaideep777/rpmodel/releases/tag/v1.0.0h), with options to use the acclimating and instantaneous responses. A C++/Rcpp version is also provided for potential integration with vegetation models (https://github.com/jaideep777/phydro). These packages provide the option to either use the semi-analytical solution derived in this work, or to directly optimize the profit function numerically. The numerical implementation also allows for quick extension of the model with different profit and cost functions. The C++ version also allows the use of an approximate calculation of *g*_s_, which substantially improves computational speed with only a minor loss of accuracy.

### Reporting summary

Further information on research design is available in the Nature Research Reporting Summary linked to this article.

## Supplementary information

Supplementary Information Supplementary text, Figs. 1–6, and Tables 1 and 2.

Reporting Summary.

Supplementary Data 1 Dataset containing meta-analysis of soil drought experiments used for model calibration and validation.

Supplementary Data 2 Dataset containing Δ*ψ* measurements from the soil drought experiments in Dataset 1.

## Data Availability

All data used in this manuscript are compiled from the literature. We have provided citations to publications and databases at appropriate locations in the manuscript. The compiled database can be found in the Supplementary Information.

## Code availability

R code to run our model (P-hydro) is provided as an extension of the ‘rpmodel’ package (the version used in this paper is archived at https://github.com/jaideep777/rpmodel/releases/tag/v1.0.0h), with options to use the acclimating and instantaneous responses. A C++ version is also provided for potential integration with existing vegetation models (https://github.com/jaideep777/phydro). The vignettes folder of this package also contains R code to reproduce key results.
