# Supplementary material for: Towards a unified theory of plant photosynthesis and hydraulics
Source: Nat Plants. 2022 Oct 27;8(11):1304–16. doi: 10.1038/s41477-022-01244-5 (PMC9663302; doi:10.1038/s41477-022-01244-5)
Supplement: Supplementary file 1 — Supplementary text, Figs. 1–6, and Tables 1 and 2. [file 41477_2022_1244_MOESM1_ESM.pdf]

nature plants

Article

https://doi.org/10.1038/s41477-022-01244-5

Towards a unified theory of plant photosynthesis and hydraulics

In the format provided by the authors and unedited

Supplementary Information:
Towards a unified theory of plant photosynthesis and hydraulics

Jaideep Joshi, Benjamin D. Stocker, Florian Hofhansl, Shuangxi Zhou,
Ulf Dieckmann, and Iain Colin Prentice

Contents

1 Model description 2
1.1 Water-transport module and water-balance principle 2
1.1.1 Water potential 2
1.1.2 Volumetric water flow 2
1.1.3 Hydraulic conductivity 2
1.1.4 Equation governing water flow 3
1.1.5 Solution of the equation governing water flow 4
1.1.6 Transpiration flow 4
1.1.7 Water-balance principle 5
1.2 Photosynthesis module and photosynthetic-coordination hypothesis 6
1.2.1 Standard biochemical model of photosynthesis 6
1.2.2 Rate of CO_2 diffusion into the leaves 7
1.2.3 CO_2 balance 7
1.2.4 Photosynthetic-coordination hypothesis 8
1.3 Optimization module and profit-maximization hypothesis 9
1.3.1 Profit-maximization hypothesis 9
1.3.2 Semi-analytical solution to the profit-maximization model 9
1.3.3 Fully analytical solution for a special case 10
2 Model extension 11
2.1 Extended water-transport module and water-balance principle 11
2.1.1 Drop in water potential across the root-outside-xylem segment 11
2.1.2 Drop in water potential across the xylem segment 11
2.1.3 Drop in water potential across the leaf outside-xylem segment 13
2.1.4 Flow continuity between the three segments 13
2.1.5 Simplified three-segment model under non-stressed conditions 13
2.1.6 Water-balance principle 14
3 Supplementary figures 15

4 Cross-validation........23
5 Sources of empirical trait values........24
6 References........24

1 Model description

Our model comprises three components specified as modules: the water-transport module (Section 1.1), the photosynthesis module (Section 1.2), and the optimization module (Section 1.3). Variables and notations for the three modules are summarized in Tables M1-M3, respectively.

1.1 Water-transport module and water-balance principle

In this section, we introduce the biophysical concepts and terminology necessary for describing plant hydraulics, describe the equation governing water flow within a plant as well as its solution, and specify and apply the water-balance principle.

1.1.1 Water potential

The water potential ψ measures the potential energy of water per unit volume, with the value $\psi = 0$ corresponding to liquid water in fully saturated soil under reference conditions. Accordingly, the water potential ψ becomes increasingly negative as water ascends through a plant's hydraulic pathway, reaching very negative values when it exits the plant into the atmosphere as water vapour through the stomata.

1.1.2 Volumetric water flow

The volumetric flow rate q of water through a pipe is given by Darcy's law,

$$q = \frac{k}{\eta}\Delta\psi,$$

where k is the conductance of the pipe to water flow depending on the pipe's cross-sectional area A and length L, η is the viscosity of water, and $\Delta\psi$ is the water-potential difference between the beginning and end of the pipe. In plant hydraulics, it is often convenient to express the water flow rate q in terms of a flow rate Q per unit surface area,

$$Q = \frac{q}{A} = \frac{K}{\eta}\Delta\psi, \quad \text{S1}$$

where $K = k/A$ is the conductance of the pipe per unit surface area. Since we follow the widespread convention of expressing Q as the flow rate per unit leaf area rather than per unit sapwood cross-sectional area, we choose to also express the conductance K per unit leaf area.

1.1.3 Hydraulic conductivity

The longer water must travel through a pipe, the more resistance – and thus, less conductance – it experiences: specifically, the conductance K is inversely proportional to the pipe length L,

$$K = \frac{\kappa}{L}. \quad \text{S2}$$

where κ is the hydraulic conductivity, which measures the permeability of the conducting medium. Since K is already expressed per unit surface area and the inverse relationship with length is accounted for as indicated above, κ is independent of all dimensions of the pipe, instead characterising the conducting medium.

In the plant, water first flows outside the xylem in the roots, then it enters the xylem and flows through xylem in roots, stem, and leaves, and then exits the xylem and flows outside of it, up to the stomata. There are two sources of variation in conductivity along the hydraulic pathway. First, conductivity depends on the internal structure of the xylem and outside-xylem tissues. This internal structure can change systematically with plant height, such as through the continuous tapering of the xylem vessels. Therefore, conductivity can be expressed as a function of the distance h from the soil-root interface (Fig. M1). Second, factors such as reduced aquaporin activity or a reduction in the permeability of the cell membranes in roots and leaves cause a decline in the outside-xylem conductivity as water potential decreases. Similarly, as water potential decreases, individual xylem conduits embolize, leading to a reduction in xylem conductivity. Thus, conductivity is also a function of water potential. Outside-xylem conductivity is typically much lower than xylem conductivity and is lost much before xylem begins to embolize. Furthermore, recovery of this conductivity is often slower than its loss, leading to hysteresis in the conductivity-water-potential relationship. Assuming that the effects of hysteresis are negligible under the range of water potentials experienced by plants before stomatal closure, the dependence on water potential can be described phenomenologically by a Weibull function,

$$\kappa(h,\psi) = \kappa_{\max}(h)P(h,\psi) = \kappa_{\max}(h)\left(\frac{1}{2}\right)^{\left(\frac{\psi}{\psi_{50}(h)}\right)^{b}},$$

where $\kappa_{\max}(\mathrm{h})$ is the maximum conductivity at distance h (i.e., the conductivity at $\psi = 0$), $P(h,\psi)$ is the so-called vulnerability curve (Eq. 2 in the main text) of a slice of the conducting segment at distance h, $\psi_{50}(\mathrm{h})$ is the water potential at which 50% conductivity is lost at distance h (i.e., $\kappa\big(h,\psi_{50}(\mathrm{h})\big) = \kappa_{\max}(\mathrm{h})/2$), and b is the vulnerability curve's shape parameter, such that a higher value of b leads to a steeper decline in conductivity around $\psi = \psi_{50}(\mathrm{h})$. Note that both $\kappa_{\max}(\mathrm{h})$ and $\psi_{50}(\mathrm{h})$ can in general depend on the distance h from the tip of the root.

1.1.4 Equation governing water flow

The drop of water potential along a hydraulic pathway's cross-section over a small distance increment $\mathrm{d}h$ occurs for two reasons, due to gravity and due to water flow. The drop in water potential due to gravity is $\rho g_{\parallel}\mathrm{d}h$ (first term on the right-hand side in the equation below), where ρ is the density of water and $g_{\parallel}$ is the component of the gravitational acceleration in the direction of the water flow. The drop due to a volumetric flow rate of liquid water per unit area, Q, through the pathway (second term on the right-hand side in the equation below) follows from Eqs. S1 and S2. Thus, the total drop in water potential is given by (Fig. M1),

$$\psi(h+\mathrm{d}h) - \psi(h) = -\rho g_{\parallel}\mathrm{d}h - \frac{Q\eta\mathrm{d}h}{\kappa\big(h,\psi(h)\big)},$$

where $\psi(h)$ is the water potential at a distance h from the plant base, $\mathrm{d}h$ is the considered small distance increment, η is the dynamic viscosity of water, and $\kappa\big(h,\psi(h)\big)$ is the conductivity at a distance h with water potential $\psi(h)$.

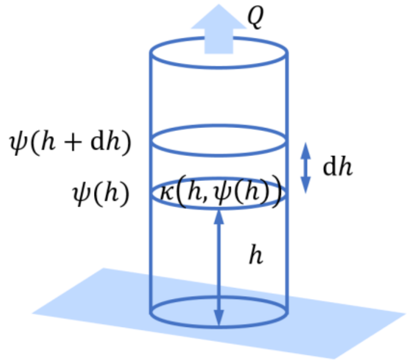

Fig. M1. Schematic illustration of the flow of water through a conducting segment.

This yields a differential equation describing the water flow through any conducting segment whose conductivity may vary with distance and water potential,

$$\frac{\mathrm{d}\psi}{\mathrm{d}h} = -\rho g_{\parallel} - \frac{Q\eta}{\kappa_{\max}(h)P(h, \psi(h))}, \quad \text{S3}$$

1.1.5 Solution of the equation governing water flow

If we ignore the effects of gravity (assuming $\rho g_{\parallel} \ll Q\eta/\kappa$) and structural variation along the pathway (i.e., assuming $\kappa_{\max}(h)$ to be independent of h, and $P(h, \psi(h))$ to depend on h only through $\psi(h)$), we can solve Eq. S3 to express Q as

$$Q = -\frac{\kappa_{\max}}{L\eta} \int_{\psi_s}^{\psi_l} P(\psi) \mathrm{d}\psi\,, \quad \text{S4}$$

where ψ_s and ψ_l are the water potentials in the soil and in the leaves (near the stomata), respectively, and L is the length of the hydraulic pathway between the soil and the stomata. The vulnerability curve $P(\psi)$ here describes the combined effect of the loss of conductivity of the xylem and outside-xylem segments.

The parameter K_p estimated in our model fits is the maximum leaf-specific whole-plant conductance, i.e., the maximum conductance of the entire plant including roots, stem, and leaves, expressed per unit leaf area. It equals the ratio $\kappa_{\max}/L$, and represents the combined effect of xylem and leaf permeabilities, plant height, and leaf thickness.

The assumption of no structural variation along the pathway is not true for xylem, because xylem tapering typically reduces the length dependence on conductivity. However, since xylem is the most conductive tissue within the plant, the maximum contribution to the term $\kappa_{\max}/L$ is from the outside-xylem pathways. In these pathways, the above derivation applies, with L being the length of the outside-xylem pathways.

1.1.6 Transpiration flow

Water exits from the leaves due to transpiration. The volumetric transpiration flow rate E of water vapour per unit leaf area is given by Fick's law of diffusion,

$$E = 1.6 g_s (e_l - e_a),$$

where g_s is the stomatal conductance to CO_2, and the factor 1.6 is the ratio of the diffusivities of water vapour and CO_2, which therefore converts g_s to the stomatal conductance to water vapour. Furthermore, e_a is the relative partial pressure of water vapour in the atmosphere, and e_l is the relative partial pressure of water vapour inside the leaf, both of which are expressed as fractions of the atmospheric pressure.

The air inside the leaf is typically saturated with water vapour, such that $e_l \approx e_s$, where e_s is the saturation vapour pressure as a fraction of the atmospheric pressure, and the difference $e_s - e_a$ is the atmospheric vapour pressure deficit D. Thus,

$$E \approx 1.6 g_s D. \qquad \text{S5}$$

Notice that, even though g_s is typically referred to as a “conductance”, its definition following from Fick’s law of diffusion is that of a diffusion coefficient, rather than that of a hydraulic conductance used for characterizing the flow of liquid water.

1.1.7 Water-balance principle

Since there is no water storage in the leaves, water balance applies. This means that the hydraulic flow rate Q at which water enters the leaves according to Eq. S4 equals the transpiration flow rate E at which water vapour diffuses out of the leaves into the atmosphere according to Eq. S5,

$$Q = E.$$

This allows us to express g_s in terms of $\Delta\psi = \psi_s - \psi_l$,

$$g_s = -\frac{K_p}{1.6 D \eta} \int_{\psi_s}^{\psi_s - \Delta\psi} P(\psi) \mathrm{d}\psi \,, \qquad \text{S6}$$

which shows that, as a consequence of water balance, g_s is independent of χ.

**Symbol**	**Meaning**	**Unit**
k	Conductance	m^3
K	Conductance per unit area	m
K_p	Maximum whole-plant conductance per unit leaf area	m
κ	Conductivity, i.e., conductance per unit area at unit length	m^2
ψ	Water potential	Pa
ψ_{50}	Water potential at which 50% conductivity is lost	Pa
ψ_s	Soil water potential	Pa
ψ_l	Leaf water potential	Pa
η	Viscosity of water	Pa s
ρ	Density of water	kg m^{-3}
$P(\psi)$	Vulnerability curve, i.e., fraction of maximum conductivity remaining at water potential ψ	–
q	Volumetric flow rate of liquid water	m^3 s^{-1}

Q	Volumetric flow rate of liquid water per unit area	m s^{-1}
E	Volumetric transpiration flow rate of water vapour per unit area	m s^{-1}
g_s	Stomatal conductance, i.e., amount of CO_2 or water vapour entering or exiting leaves per unit leaf area per unit concentration gradient	mol m^{-2} s^{-1}
e_a	Partial pressure of water vapour in the atmosphere divided by atmospheric pressure	–
e_l	Partial pressure of water vapour inside the leaf divided by atmospheric pressure	–
e_s	Saturation vapour pressure divided by atmospheric pressure	–
D	Vapour pressure deficit divided by atmospheric pressure	–

Table M1. Variables and notations in the water-transport module.

1.2 Photosynthesis module and photosynthetic-coordination hypothesis

In this section, we describe the standard biochemical model of photosynthesis, also called the Farquhar-von Caemmerer-Berry biochemical model, and specify and apply the photosynthetic-coordination hypothesis.

1.2.1 Standard biochemical model of photosynthesis

Photosynthesis is the process by which plant leaves use light energy to convert CO_2 from the atmosphere and water from the soil into sugars. It depends on two sequential reactions: (1) the light-dependent electron-transport reaction, through which photosystems in the leaf use light energy to strip electrons from molecules of water and use them to produce NADPH and ATP molecules, which act as short-term energy reserves, and (2) the light-independent carboxylation reaction, through which NADPH and ATP molecules are used to power the Calvin cycle, in which CO_2 is absorbed with the help of an enzyme called RuBisCO and fixed into sugars. Thus, photosynthesis is jointly limited by light availability (which is further subject to the efficiency of the photosystems) and the carboxylation capacity of leaves (i.e., the chemical activity of RuBisCO).

The carboxylation-limited rate A_c of photosynthesis is given by the Farquhar-von Caemmerer-Berry biochemical model [47] ,

$$A_c = V_{\mathrm{cmax}} \frac{c_i - \Gamma^*}{c_i + K_M} - R_d, \quad \text{S7}$$

where V_{cmax} is the maximum carboxylation capacity of leaves, c_i is the leaf-internal CO_2 concentration, Γ^* is the photosynthetic light-compensation point, K_M is the Michaelis-Menten coefficient for C3 photosynthesis, and R_d is the dark-respiration rate, which is assumed to be proportional to V_{cmax},

$$R_d = b_r V_{\mathrm{cmax}},$$

where b_r is the dark-respiration rate per unit carboxylation capacity. The quantities c_i , Γ^*, and K_M are all expressed as partial pressures divided by the atmospheric pressure and are thus unitless.

The light-limited rate A_j of photosynthesis is also given by the Farquhar-von Caemmerer-Berry biochemical model,

$$A_\mathrm{j} = \frac{J}{4}\left(\frac{c_\mathrm{i} - \Gamma^*}{c_\mathrm{i} + 2\Gamma^*}\right) - R_\mathrm{d}, \qquad \text{S8}$$

where J is the effective electron-transport capacity of the leaves, jointly determined by the light availability I_abs and the leaves' intrinsic maximum electron-transport capacity J_max. The factor '4' accounts for the fact that for every four photons absorbed by the leaf's photosystems, one electron is released. The response of J to light availability is saturating, phenomenologically expressed by a rectangular hyperbola,

$$J = \frac{4\phi_0 I_\mathrm{abs}}{\sqrt{\left(\frac{4\phi_0 I_\mathrm{abs}}{J_\mathrm{max}}\right)^2 + 1}}, \qquad \text{S9}$$

where J_max is the maximum electron-transport capacity of leaves (approached when $I_\mathrm{abs} \to \infty$), ϕ_0 is the intrinsic quantum-yield efficiency, and I_abs is the incident photosynthetic photon-flux density, measuring light availability in terms of the absorbed photosynthetically active solar radiation.

Inverting the above relation, we can express J_max in terms of J,

$$J_\mathrm{max} = \frac{4\phi_0 I_\mathrm{abs}}{\sqrt{\left(\frac{4\phi_0 I_\mathrm{abs}}{J}\right)^2 - 1}}. \qquad \text{S10}$$

Finally, since the carboxylation reaction follows electron-transport reaction, the rate A_p of photosynthesis is limited by the slower of the two reactions,

$$A_\mathrm{p} = \min(\mathrm{A_c}, \mathrm{A_j}).$$

1.2.2 Rate of CO_2 diffusion into the leaves

The rate A_d at which CO_2 diffuses from the atmosphere into the leaves is again given by Fick's law,

$$A_\mathrm{d} = g_\mathrm{s}(c_\mathrm{a} - c_\mathrm{i}) = g_\mathrm{s} c_\mathrm{a}(1 - \chi), \qquad \text{S11}$$

where χ is the ratio $c_\mathrm{i}/c_\mathrm{a}$ of the leaf-internal and ambient CO_2 partial pressures.

1.2.3 CO_2 balance

Under equilibrium conditions, there is no storage or build-up of CO_2 in the leaves. Therefore, the rate of photosynthesis, i.e., the rate at which CO_2 is fixed by the leaves, equals the rate at which CO_2 diffuses into the leaves,

$$A_\mathrm{d} = A_\mathrm{p}.$$

1.2.4 Photosynthetic-coordination hypothesis

The photosynthetic-coordination hypothesis (see main text) states that, under typical daytime conditions, the photosynthetic capacities are modulated by leaves such that the carboxylation-limited and light-limited assimilation rates are equal,

$$A_\mathrm{c} = A_\mathrm{j}.$$

Therefore,

$$V_\mathrm{cmax}\frac{c_\mathrm{i} - \Gamma^*}{c_\mathrm{i} + K_\mathrm{M}} - R_\mathrm{d} = \frac{J}{4}\left(\frac{c_\mathrm{i} - \Gamma^*}{c_\mathrm{i} + 2\Gamma^*}\right) - R_\mathrm{d},$$

which gives

$$V_\mathrm{cmax} = \frac{J}{4}\left(\frac{c_\mathrm{i} + K_\mathrm{M}}{c_\mathrm{i} + 2\Gamma^*}\right). \quad \text{S12}$$

Hence, A_j can be rewritten as

$$A_\mathrm{j} = \frac{J}{4}\cdot\frac{c_\mathrm{i}(1 - b_\mathrm{r}) - (\Gamma^* + b_\mathrm{r}K_\mathrm{M})}{c_\mathrm{i} + 2\Gamma^*}. \quad \text{S13}$$

Equating Eq. S11 and Eq. S13, J can be expressed in terms of χ as

$$J = 4g_\mathrm{s}c_\mathrm{a}\frac{(1-\chi)(\chi c_\mathrm{a} + 2\Gamma^*)}{\chi c_\mathrm{a}(1 - b_\mathrm{r}) - (\Gamma^* + b_\mathrm{r}K_\mathrm{M})}. \quad \text{S14}$$

By substituting Eq. S14 into Eq. S10, we obtain an expression for J_max in terms of g_s and χ.

Symbol	Meaning	Unit
A_c	Carboxylation-limited photosynthesis rate	mol m^{-2} s^{-1}
A_j	Electron-transport-limited photosynthesis rate	mol m^{-2} s^{-1}
A_p	Rate of photosynthesis, i.e., rate of CO_2 fixation in the leaves	mol m^{-2} s^{-1}
A_d	Rate at which CO_2 diffuses from the atmosphere into the leaves	mol m^{-2} s^{-1}
c_i	Leaf-internal CO_2 partial pressure, divided by atmospheric pressure	–
c_a	Ambient CO_2 partial pressure, divided by atmospheric pressure	–
χ	Ratio of c_i and c_a	–
Γ^*	Photosynthetic light-compensation point	–
K_M	Michaelis-Menten coefficient for C3 photosynthesis	–
V_cmax	Maximum carboxylation capacity of leaves	mol m^{-2} s^{-1}
J	Effective electron-transport capacity of leaves	mol m^{-2} s^{-1}
J_max	Maximum electron-transport capacity of leaves, under light saturation	mol m^{-2} s^{-1}
R_d	Dark-respiration rate	mol m^{-2} s^{-1}
ϕ_0	Intrinsic quantum-yield efficiency	–
I_abs	Incident photosynthetic photon-flux density	mol m^{-2} s^{-1}
b_r	Dark-respiration rate per unit carboxylation capacity	–

Table M2. Variables and notations in the photosynthesis module.

1.3 Optimization module and profit-maximization hypothesis

In this section, we specify and apply the profit-maximization hypothesis and describe our semi-analytical solution to the resulting optimality problem, as well as a useful special case possessing a simple analytical solution.

1.3.1 Profit-maximization hypothesis

We assume that plants maximize their profit F defined as the benefit from photosynthetic assimilation minus the costs of maintaining the photosynthetic capacities and the hydraulic pathway,

$$F = A_\mathrm{j} - \alpha J_\mathrm{max} - \gamma \Delta\psi^2,$$

where each term depends on at least one of the two independent variables χ and $\Delta\psi$,

$$F(\chi, \Delta\psi) = g_\mathrm{s}(\Delta\psi) c_\mathrm{a}(1-\chi) - \alpha J_\mathrm{max}(\chi, \Delta\psi) - \gamma \Delta\psi^2.$$

1.3.2 Semi-analytical solution to the profit-maximization model

To solve the optimality condition, we set the gradient of the profit function to zero,

$$\begin{aligned} \frac{\partial F}{\partial \chi} &= -g_\mathrm{s} c_\mathrm{a} - \alpha \frac{\partial J_\mathrm{max}}{\partial J}\frac{\partial J}{\partial \chi} - 0 = 0, \\ \frac{\partial F}{\partial \Delta\psi} &= \frac{\partial g_\mathrm{s}}{\partial \Delta\psi} c_\mathrm{a}(1-\chi) - \alpha \frac{\partial J_\mathrm{max}}{\partial J}\frac{\partial J}{\partial \Delta\psi} - 2\gamma\Delta\psi = 0. \end{aligned} \quad \text{S15}$$

The four derivatives required in the above equations are as follows,

$$\begin{aligned} \frac{\partial J_\mathrm{max}}{\partial J} &= \frac{(4\phi_0 I_\mathrm{abs})^3}{((4\phi_0 I_\mathrm{abs})^2 - J^2)^{3/2}}, \\ \frac{\partial J}{\partial \chi} &= 4 g_\mathrm{s} c_\mathrm{a} \left(b_\mathrm{r} \frac{2\frac{\Gamma^*}{c_\mathrm{a}}\left(\frac{K_\mathrm{M}}{c_\mathrm{a}}+1\right) + \frac{K_\mathrm{M}}{c_\mathrm{a}}(2\chi-1) + \chi^2}{\left(b_\mathrm{r}\left(\frac{K_\mathrm{M}}{c_\mathrm{a}}+\chi\right) + \frac{\Gamma^*}{c_\mathrm{a}} - \chi\right)^2} \right. \\ &\qquad \left. - \frac{(\chi - \frac{\Gamma^*}{c_\mathrm{a}})^2 + 3\frac{\Gamma^*}{c_\mathrm{a}}(1-\frac{\Gamma^*}{c_\mathrm{a}})}{\left(b_\mathrm{r}\left(\frac{K_\mathrm{M}}{c_\mathrm{a}}+\chi\right) + \frac{\Gamma^*}{c_\mathrm{a}} - \chi\right)^2} \right), \\ \frac{\partial J}{\partial \Delta\psi} &= 4 \frac{\partial g_\mathrm{s}}{\partial \Delta\psi} c_\mathrm{a} \frac{(1-\chi)(\chi c_\mathrm{a} + 2\Gamma^*)}{\chi c_\mathrm{a}(1-b_\mathrm{r}) - (\Gamma^* + b_\mathrm{r} K_\mathrm{M})}, \\ \frac{\partial g_\mathrm{s}}{\partial \Delta\psi} &= \frac{\partial}{\partial \Delta\psi}\left(-\frac{K_\mathrm{L}}{1.6 D\eta} \int_{\psi_\mathrm{s}}^{\psi_\mathrm{s}-\Delta\psi} P(\psi)\mathrm{d}\psi \right) = \frac{K_\mathrm{L}}{1.6 D\eta} P(\psi_\mathrm{s} - \Delta\psi). \end{aligned} \quad \text{S16}$$

By substituting the derivatives in Eq. S16 into Eq. S15, we obtain two equations involving the unknowns χ and $\Delta\psi$, which could be solved using a two-dimensional root-finding algorithm.

However, Eq. S15 can be simplified analytically: to this end, we first rearrange both equations in Eq. S15 to move $\partial J_{\max}/\partial J$ to their left-hand sides and then eliminate this term by equating the resultant right-hand sides,

$$-\frac{g_s c_a}{\alpha \frac{\partial J}{\partial \chi}} = \frac{g_s'(\Delta\psi) c_a (1-\chi) - 2\gamma \Delta\psi}{\alpha \frac{\partial J}{\partial \Delta\psi}}. \qquad \text{S17}$$

This equation yields χ in terms of $\Delta\psi$,

$$\chi^*(\Delta\psi) = \Big(c_a^2\big((3-2b_r)\Gamma^* + b_r K_M\big) g_s'(\Delta\psi) - 2c_a \Delta\psi\gamma(b_r K_M + \Gamma^*) - \sqrt{2c_a^2 \Delta\psi\gamma\big((2b_r-3)\Gamma^* - b_r K_M\big)\big((b_r-1)c_a + b_r K_M + \Gamma^*\big)\big((c_a + 2\Gamma^*)g_s'(\Delta\psi) - 2\Delta\psi\gamma\big)} \Big) \Big/ \Big(c_a^2 \Big(\big((3-2b_r)\Gamma^* + b_r K_M\big) g_s'(\Delta\psi) + 2(b_r - 1)\Delta\psi\gamma \Big) \Big).$$

Substituting this solution for χ into Eq. S15 results in all terms to depend on $\Delta\psi$ alone. The optimal $\Delta\psi^*$ can then be found using a one-dimensional root-finding algorithm, instead of solving Eqs. S13 and S14 using a more complex two-dimensional root-finding algorithm.

1.3.3 Fully analytical solution for a special case

In the special case of strong electron-transport limitation or, equivalently, high light availability, the optimal χ can be obtained analytically, and turns out to be independent of $\Delta\psi$: $J_{\max} \ll 4\phi_0 I_{\text{abs}}$ implies $J \approx J_{\max}$ and thus

$$\frac{\partial J_{\max}}{\partial J} = 1,$$

from which we obtain

$$\frac{\partial F}{\partial \chi} = -g_s c_a - \alpha \frac{\partial J}{\partial \chi} = 0.$$

This is a quadratic equation in χ, giving

$$\chi^* = \frac{(1-4\alpha-b_r)\left(b_r K_M + \frac{\Gamma^*}{c_a}\right) + \sqrt{4\alpha(1-4\alpha-b_r)\left(3\frac{\Gamma^*}{c_a} - b_r\left(2\frac{\Gamma^*}{c_a} + K_M\right)\right)\left(1 - \frac{\Gamma^*}{c_a} - b_r(1+K_M)\right)}}{(1-b_r)(1-4\alpha-b_r)}. \qquad S18$$

In the absence of dark respiration ($b_r = 0$), this simplifies to

$$\chi^* = \frac{\frac{\Gamma^*}{c_a}(1-4\alpha) + \sqrt{12\alpha(1-4\alpha)\frac{\Gamma^*}{c_a}\left(1 - \frac{\Gamma^*}{c_a}\right)}}{1-4\alpha}.$$

Symbol	Meaning	Unit
F	Plant profit from photosynthesis	mol m^{-2} s^{-1}
α	Unit cost of photosynthetic capacity	–
γ	Unit cost of maintaining the hydraulic pathway	mol m^{-2} s^{-1} Pa^{-2}

Table M3. Variables and notations in the optimization module.

2 Model extension

In the present study, we use an unsegmented water-transport model, in which the entire hydraulic pathway is represented by a single effective segment, as shown in Section 1.1. To facilitate future research, we also derive and present an extended three-segment version of the water-transport model that can explicitly account for the hydraulic properties of the roots, stem, and the leaves, as shown in Fig. 1.

2.1 Extended water-transport module and water-balance principle

To derive the total drop in water potential from the soil to the air, we must find and add the drops in water potential along the root-outside-xylem, xylem (in the roots, stem, and leaves), and leaf-outside-xylem segments.

2.1.1 Drop in water potential across the root-outside-xylem segment

Water first enters the plant via roots by crossing the soil-root interface. To reach the xylem, it must flow through the cells of the root cortex, the endodermis, and the stele, either via cell walls and intercellular spaces (apoplastic pathway) or through cell cytoplasm and plasmodesmata (symplastic pathway). This outside-xylem segment in the root often offers much larger resistance to water flow compared to the xylem segment and, as water potential decreases, it loses conductivity much faster than the xylem segment.

We can represent the total flow Q_r through different fine-root hydraulic structures (including the cell walls and the cell mesophyll) as

$$Q_\mathrm{r} = -\frac{\kappa_\mathrm{r}}{L_\mathrm{r}\eta}\int_{\psi_\mathrm{s}}^{\psi_\mathrm{r}} P_\mathrm{r}\,(\psi)\mathrm{d}\psi, \qquad \text{S19}$$

where κ_r is the conductivity of the root system (including the soil-root interface) per unit leaf area, ψ_r is the water potential at the end of the pathway (i.e., at the beginning of the xylem), and L_r is the length of the hydraulic pathway outside the xylem. The conductivity $\kappa_\mathrm{r} = \hat{\kappa}_\mathrm{r}\zeta$ may be linked to the fine-root mass per unit leaf area, ζ, where $\hat{\kappa}_\mathrm{r}$ is the conductivity of the root system per unit fine-root mass.

2.1.2 Drop in water potential across the xylem segment

The Hagen-Poiseuille equation describes the pressure drop $\Delta\psi$ along a cylindrical pipe of length L and radius R due to water flowing in it at the rate q,

$$\Delta\psi = \frac{8\eta L q}{\pi R^4},$$

where η is the dynamic viscosity of water.

Water is conducted through the xylem in roots, stem, and leaf veins by interconnected xylem vessels. Using Darcy's law as stated in Section 1.1.2, the conductance k_v of a single cylindrical vessel of length l_v and radius r_v thus equals

$$k_\mathrm{v} = \frac{\eta q}{\Delta\psi} = \frac{\pi r_\mathrm{v}^4}{8 l_\mathrm{v}}.$$

If the sapwood has a vessel density ρ_v, measuring the number of parallel vessels per unit sapwood area, vessel conductances will add up such that the conductivity κ_s of the sapwood xylem is given by

$$\kappa_\mathrm{s} = k_\mathrm{v} l_\mathrm{v} \rho_\mathrm{v} = \frac{\pi \rho_\mathrm{v} r_\mathrm{v}^4}{8}.$$

For example, in *Austromyrtus bidwillii*, $\rho_\mathrm{v} = 217\ \mathrm{mm}^{-2}$ and $r_\mathrm{v} = 15.25\ \mu\mathrm{m}$[81], giving $\kappa_\mathrm{s} = 4.6 \times 10^{-12}\ \mathrm{m}^2$. The conductance of the xylem segment per unit sapwood area at the site of vessel measurements is given by $K_\mathrm{s} = k_\mathrm{v}\rho_\mathrm{v} = \kappa_\mathrm{s}/l_\mathrm{v}$. Due to xylem vessel tapering, sapwood permeability typically increases from the plant tip to plant base, and has an effect of reducing the length dependence on total xylem stem conductance.

The ratio of sapwood area to leaf area is known as the Huber value ν_H. When applying Eq. S3 to plants, it is convenient to define Q as the rate of volumetric water flow per unit leaf area rather than per unit sapwood area. Therefore, we multiply the sapwood conductivity κ_s by ν_H to obtain the sapwood conductivity per unit leaf area.

Again, ignoring the effects of gravity, but accounting for xylem vessel tapering, we can derive the water flux Q_x in the xylem-segment from Eq. S3. The West-Brown-Enquist metabolic scaling theory[82] implies that sapwood permeability should increase slightly superlinearly with distance d from the tip of the plant, e.g.,

$$\kappa(d) = \kappa_\mathrm{pe} d^{1+\sigma},$$

Where κ_pe is the permeability of the petiole and σ is a small positive constant. We can then solve Eq. S3 as

$$\int_{\psi_\mathrm{r}}^{\psi_\mathrm{x}} P_\mathrm{x}(\psi)d\psi = \int_{L_\mathrm{pe}}^{L_\mathrm{x}} \frac{\eta Q_\mathrm{x}}{\kappa_\mathrm{pe}\nu_\mathrm{H} d^{1+\sigma}} dd\,,$$
$$= -\frac{\eta Q_\mathrm{x}}{\kappa_\mathrm{pe}\nu_\mathrm{H}\sigma}\left[L_\mathrm{x}^{-\sigma} - L_\mathrm{pe}^{-\sigma}\right]$$

where ψ_r and ψ_x are the water potentials at the beginning and end of the xylem segment respectively, L_x is the length of the xylem pathway (to which plant height is the key contributor), L_pe is the length of the petiole, and P_x is the vulnerability curve of the xylem, in which the loss of conductivity occurs largely through cavitation. For simplicity, we have ignored the variation in $\psi_{50\mathrm{x}}$ along the pathway. For large L_x, this gives an expression for Q_x independent of plant height,

$$Q_\mathrm{x} \approx -\frac{\kappa_\mathrm{pe}\sigma L_\mathrm{pe}^{\sigma}\nu_\mathrm{H}}{\eta}\int_{\psi_\mathrm{r}}^{\psi_\mathrm{x}} P_\mathrm{x}(\psi)d\psi. \qquad \text{S20}$$

If $\sigma = 0$, the length dependence of conductivity is not completely eliminated, but reduced from linear to logarithmic,

$$\int_{\psi_r}^{\psi_x} P_x(\psi) d\psi = \int_{L_{pe}}^{L_x} \frac{\eta Q_x}{\kappa_{pe} \nu_H d} dd,$$
$$= \frac{\eta Q_x}{\kappa_{pe} \nu_H} \log\left(\frac{L_x}{L_{pe}}\right).$$

2.1.3 Drop in water potential across the leaf outside-xylem segment

Once the water exits the xylem at the end of the leaf veins, it passes through bundle sheath cells and several layers of spongy mesophyll cells before reaching the stomata, from where it vaporizes from the cell walls and out into the atmosphere. Again, this outside-xylem segment offers much larger resistance to water flow than the xylem segment and loses conductivity much faster than the xylem segment as water potential decreases.

We can represent the total flow Q_l through all different outside-xylem leaf hydraulic structures (including the cell walls and the cell mesophyll) in the same way as for roots,

$$Q_l = -\frac{\kappa_l}{L_l \eta} \int_{\psi_x}^{\psi_l} P_l(\psi) \mathrm{d}\psi, \quad \text{S21}$$

where κ_l is the conductivity of leaves per unit leaf area, ψ_l is the water potential in the leaves (near the stomata), and L_l is the length of the leaf hydraulic pathway outside the xylem, which depends on leaf thickness and leaf venation density.

2.1.4 Flow continuity between the three segments

Since there is no loss or storage of water at the interface of any of the segments of the hydraulic pathway, the rate of water flow in the three segments is equal, $Q = Q_r = Q_x = Q_l$. Substituting these rates according to Eqs. S19-S21, we obtain

$$Q = -\frac{\kappa_r}{L_r \eta} \int_{\psi_s}^{\psi_r} P_r(\psi) \mathrm{d}\psi = -\frac{K_s \nu_H}{\eta} \int_{\psi_r}^{\psi_x} P_x(\psi) \mathrm{d}\psi = -\frac{\kappa_l}{L_l \eta} \int_{\psi_x}^{\psi_l} P_l(\psi) \mathrm{d}\psi, \quad \text{S22}$$

where K_s is the total sapwood conductance ($K_s = \kappa_{pe} \sigma L_{pe}^{\sigma}$ or $\kappa_{pe}/\log(L_x/L_{pe})$, as demonstrated in the previous section). For a given total $\Delta\psi = \psi_s - \psi_l$, Eq. S22 can be solved for ψ_x and ψ_r, and either of the three equal expressions in Eq. S22 can then be used to obtain Q.

2.1.5 Simplified three-segment model under non-stressed conditions

Since xylem conductivity is typically much higher than the outside-xylem segments and the xylem is almost never cavitated under normal operating conditions, we may assume that the potential drop across the xylem ($\Delta\psi_x = \psi_r - \psi_x$) is much smaller than that across the two outside xylem segments ($\Delta\psi_r = \psi_s - \psi_r$ and $\Delta\psi_l = \psi_x - \psi_l$). Thus, $\Delta\psi_x \approx 0$ implies that $\psi_r \approx \psi_x$. The xylem water potential can then be calculated by solving

$$Q = -\frac{\kappa_r}{L_r \eta} \int_{\psi_s}^{\psi_x} P_r(\psi) \mathrm{d}\psi = -\frac{\kappa_l}{L_l \eta} \int_{\psi_x}^{\psi_l} P_l(\psi) \mathrm{d}\psi, \quad \text{S23}$$

2.1.6 Water-balance principle

As in Section 1.1.7, since there is no water storage in the leaves, water balance applies. This means that the hydraulic flow rate Q at which water enters the leaves according to Eq. S22 equals the transpiration flow rate E at which water vapour diffuses out of the leaves into the atmosphere according to Eq. S5,

$$Q = E = 1.6 g_{\mathrm{s}} D,$$

from which g_{s} can be calculated.

Symbol	Meaning	Unit
k_{v}	Mean conductance of a single xylem vessel	m^3
r_{v}	Mean radius of a single xylem vessel	m
l_{v}	Length of a single xylem vessel	m
ρ_{v}	Number of xylem vessels per unit sapwood area	m^{-2}
κ_{s}	Conductivity of sapwood, expressed per unit sapwood area	m^2
κ_{l}	Conductivity of leaf-outside-xylem segment per unit leaf area	m^2
κ_{r}	Conductivity of root-outside-xylem segment per unit leaf area	m^2
ν_{H}	Ratio of sapwood area to leaf area, i.e., Huber value	–
L_{x}	Path length of the xylem segment, primarily driven by plant height	m
L_{l}	Path length of the leaf-outside-xylem segment	m
L_{r}	Path length of the root-outside-xylem segment	m
ψ_{p}	Water potential at the end of the xylem segment	Pa
Q	Volumetric flow rate of liquid water through the plant per unit leaf area	$m\ s^{-1}$
$\Delta\psi_{\mathrm{x}}$	Drop in water potential across the xylem segment	Pa
$\Delta\psi_{\mathrm{l}}$	Drop in water potential across the leaf-outside-xylem segment	Pa
$\Delta\psi_{\mathrm{r}}$	Drop in water potential across the root-outside-xylem segment	Pa

Table M4. Variables and notations in the extended water-transport module, beyond those already mentioned in Table M1.

3 Supplementary figures

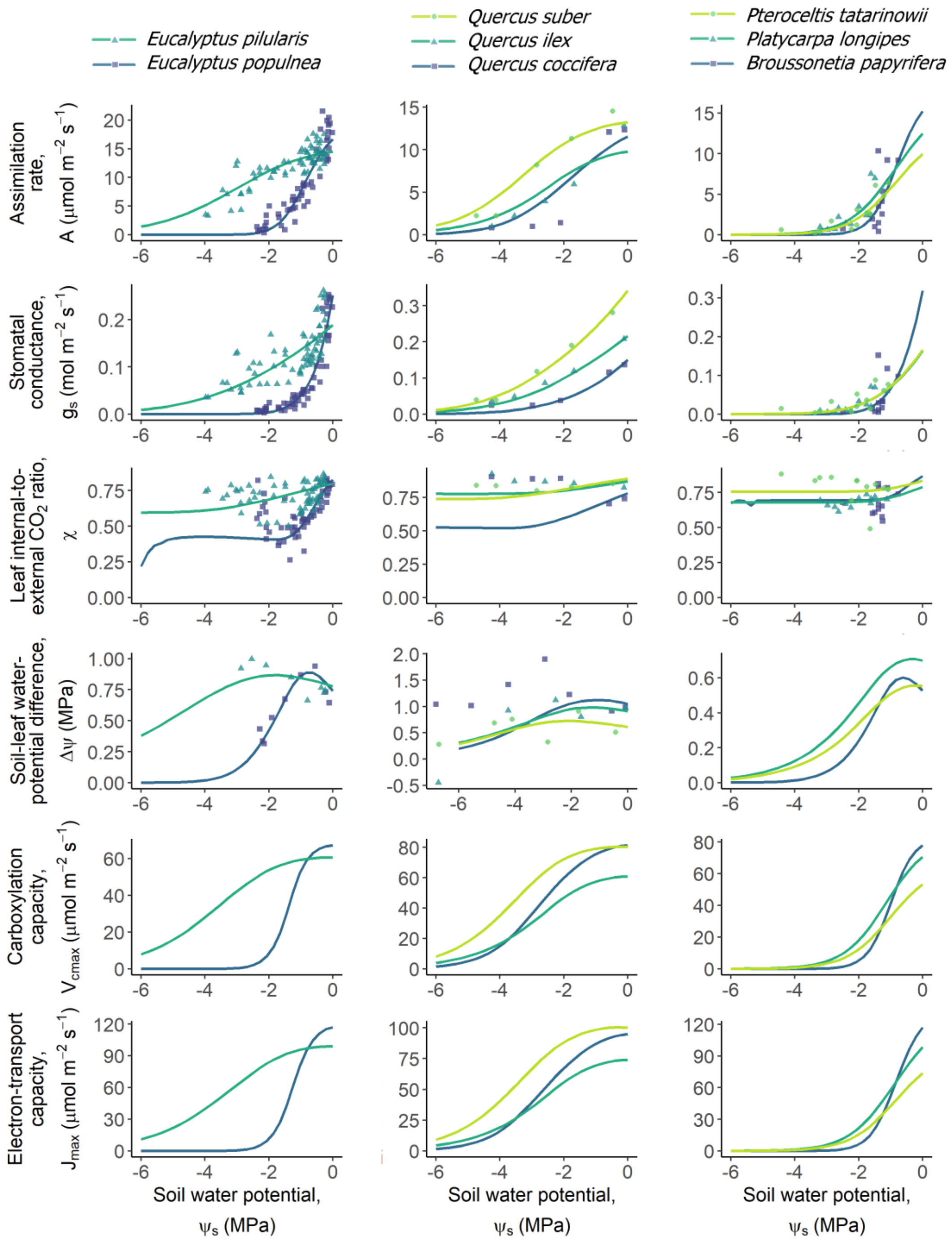

Fig. S1. Model-predicted and observed responses of the 18 analysed species to soil dry-down. Our model predictions (lines) are compared to observations (points) for all 18 species covered by our analysis. The first column in this figure reproduces Fig. 3 from the main text showing this comparison for two of the 18 species, and the comparisons for all other 16 species are shown analogously in the further columns. Each column represents a grouping of functionally similar species and has its own colour key: the different species indicated by the colours are shown, separately, at the top of each column. The last two rows do not show data points as direct measurements of photosynthetic capacities are not available; the main text provides details on our indirect validation of these predictions.

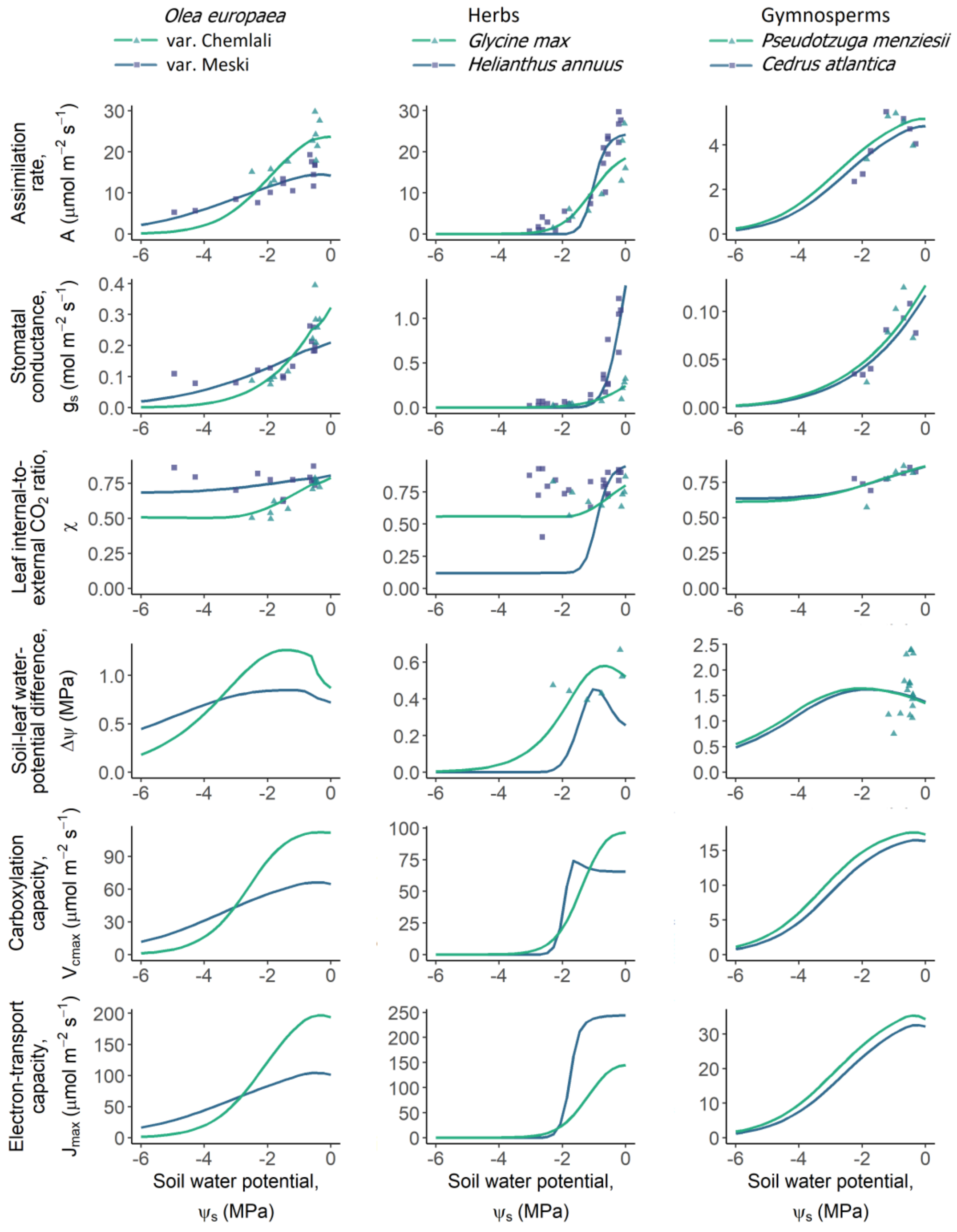

Fig. S1 (continued).

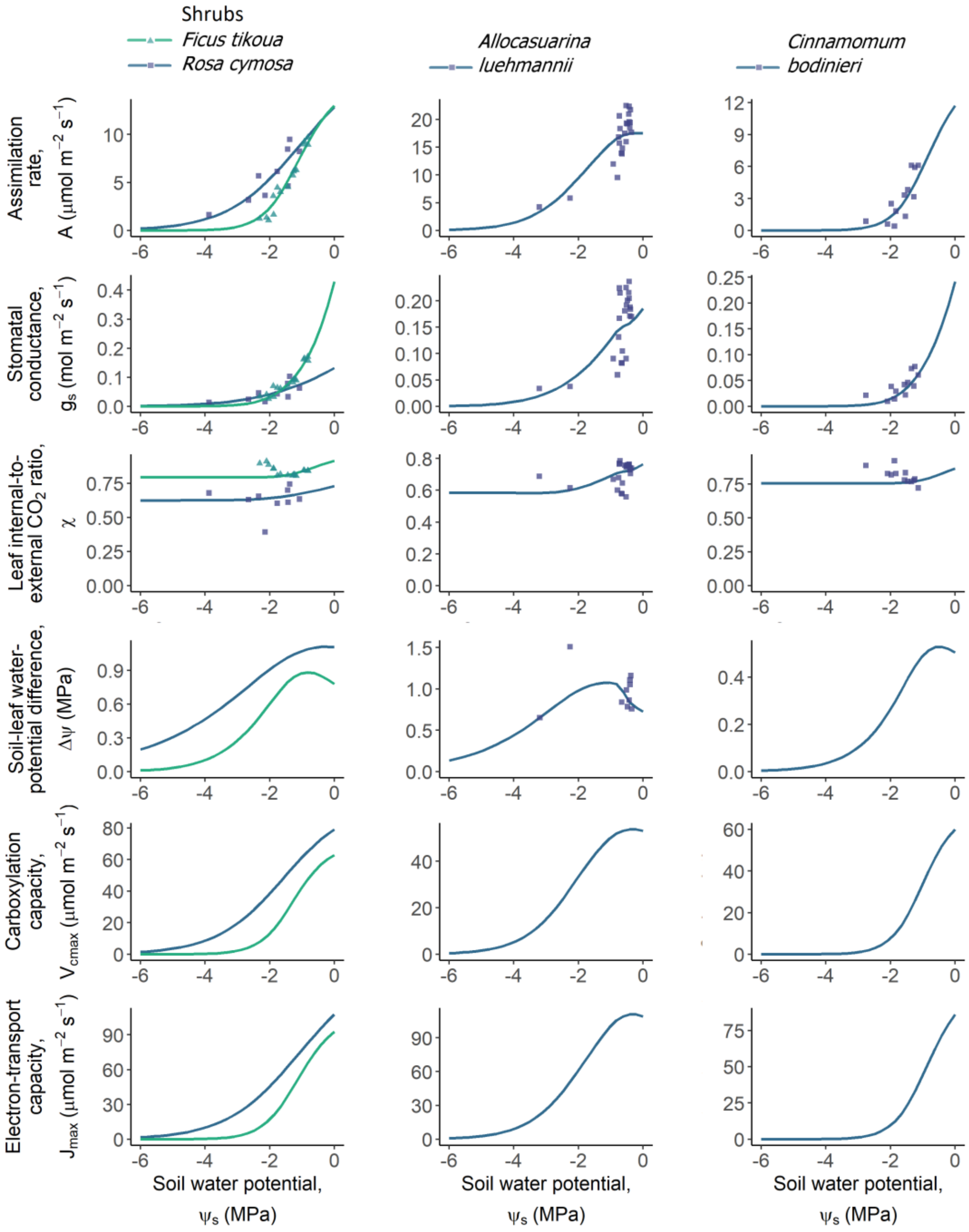

Fig. S1 (continued).

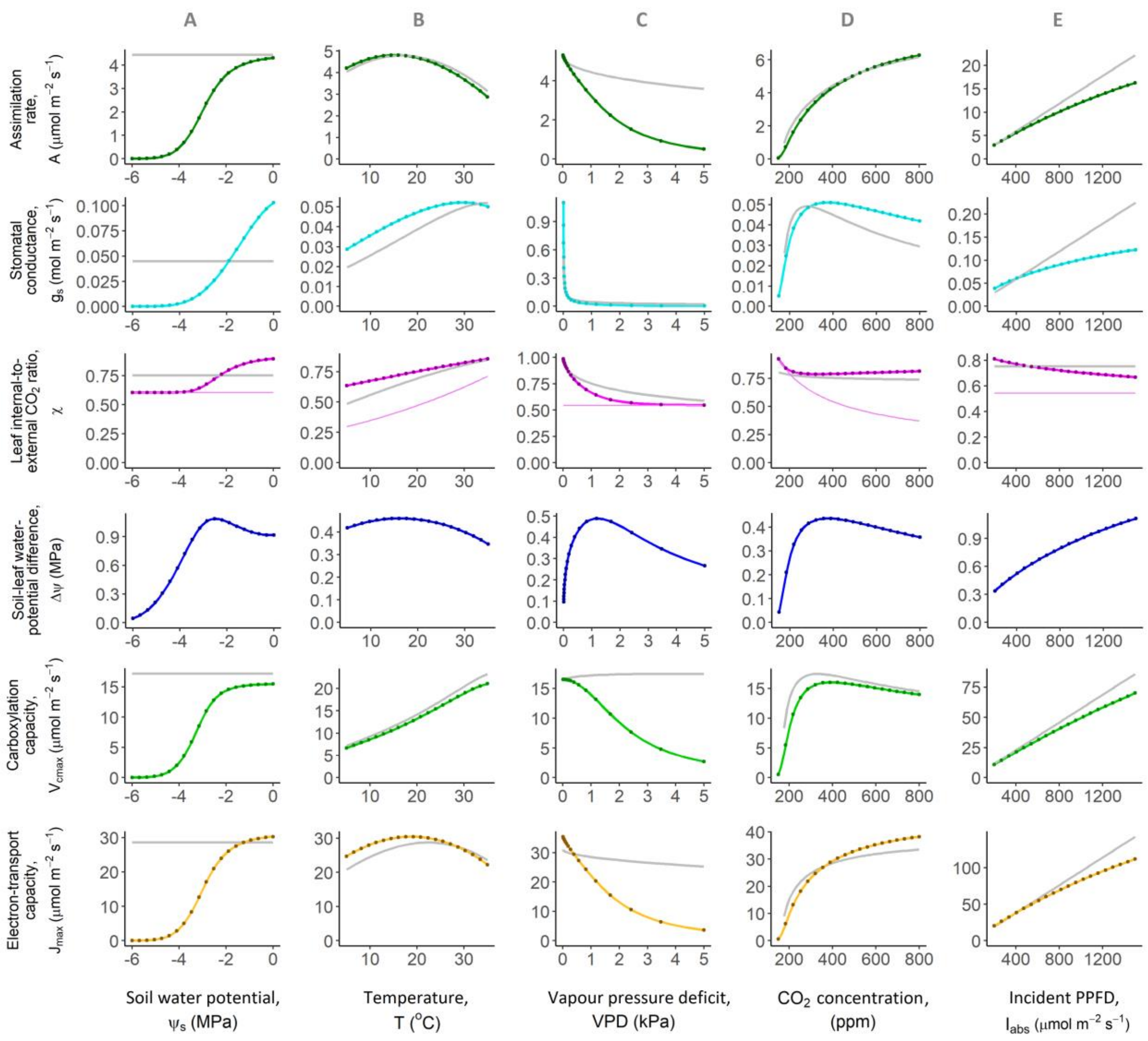

Fig. S2. Model-predicted responses to atmospheric and soil variables closely resemble those of Wang et al. (2017). Photosynthetic responses to atmospheric and soil variables as predicted by our model are shown by coloured lines and compared with those of Wang et al. (2017) shown by grey lines. Here, PPFD is the incident photosynthetic photon-flux density, i.e., light intensity. The model by Wang et al. (2017) lacks an explicit representation of hydraulics (hence no grey lines in the third row) and uses only a single universal cost parameter β. To facilitate the comparison of our model with theirs, we set $\beta = 146$ in their model and parameterize our model for a plant with the following average parameters: $K_{\mathrm{L}} = 0.3 \times 10^{-16}$ m^2, $\psi_{50} = -2$ MPa, $b = 2$, $\alpha = 0.1$, and $\gamma = 4$. Our model's predictions are shown as obtained from the semi-analytical solution to our model as described in SI Section 1.3.2 (lines) as well as from an independent numerical solution based on numerically maximizing the profit function (points). The accurate match between these semi-analytical and numerical solutions further confirms our numerical results. Thin coloured lines in the fourth row represent the $J_{\max}$-limited value of χ determined by Eq. S18.

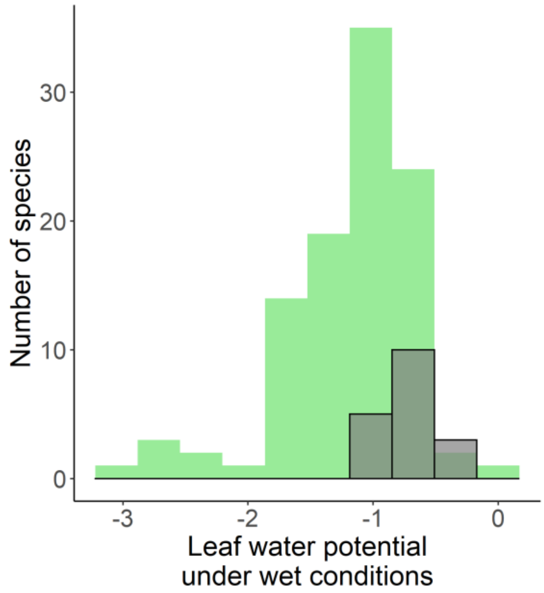

Fig. S3. Model-predicted and observed frequency distributions of leaf water potentials under wet (well-watered) conditions.Fig. 5A in the main text showed the predicted and observed distributions of the degree of anisohydricity (slope of the $\psi_l \sim \psi_s$ relationship at $\psi_s = 0$). Similarly, this figure shows the predicted (grey) and observed (green) distributions of the intercept of this relationship, i.e., the leaf water potential under wet conditions. The predicted frequency distribution is broadly consistent with the corresponding distribution observed in global empirical data obtained from Martinez-Vilalta et al. (2014).

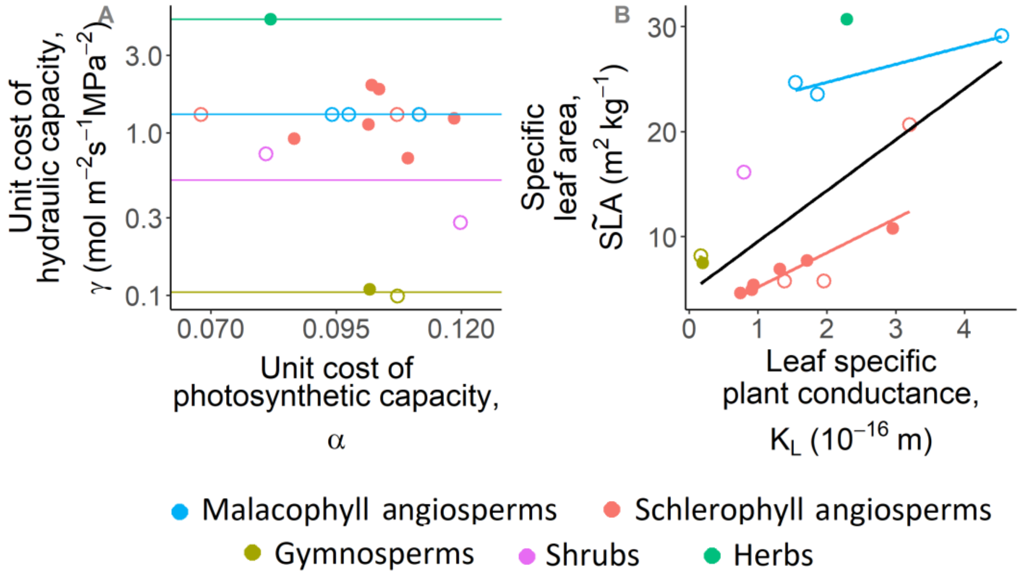

Fig. S4. Possible simplifications in setting model parameters for global model applications. (A) The unit costs α of photosynthetic capacity of the 18 analysed species vary within a narrow range, with a value of about 0.1 aptly characterizing most species. Therefore, in global model applications α could be treated as a constant. The unit costs γ of maintaining the hydraulic pathway vary lesser within, than among, plant functional types. Therefore, in global model applications γ could be treated as constants specific to plant functional types. (B) Leaf conductivity is expected to scale with inverse leaf thickness, and therefore SLA. Indeed, we find that whole-plant conductance K_p, which has a substantial contribution from leaves, is positively correlated with the specific leaf area $S\tilde{L}A$, and this correlation is stronger within than among plant functional types. Therefore, in global model applications K_p could be parameterized using widely available data on specific leaf area in conjunction with linear regressions specific to plant functional types. Closed circles indicate species for which γ was estimated using data on $\Delta\psi$, whereas open circles refer to species for which such data was not available and for which we therefore used an average value of γ estimated for the respective plant types.

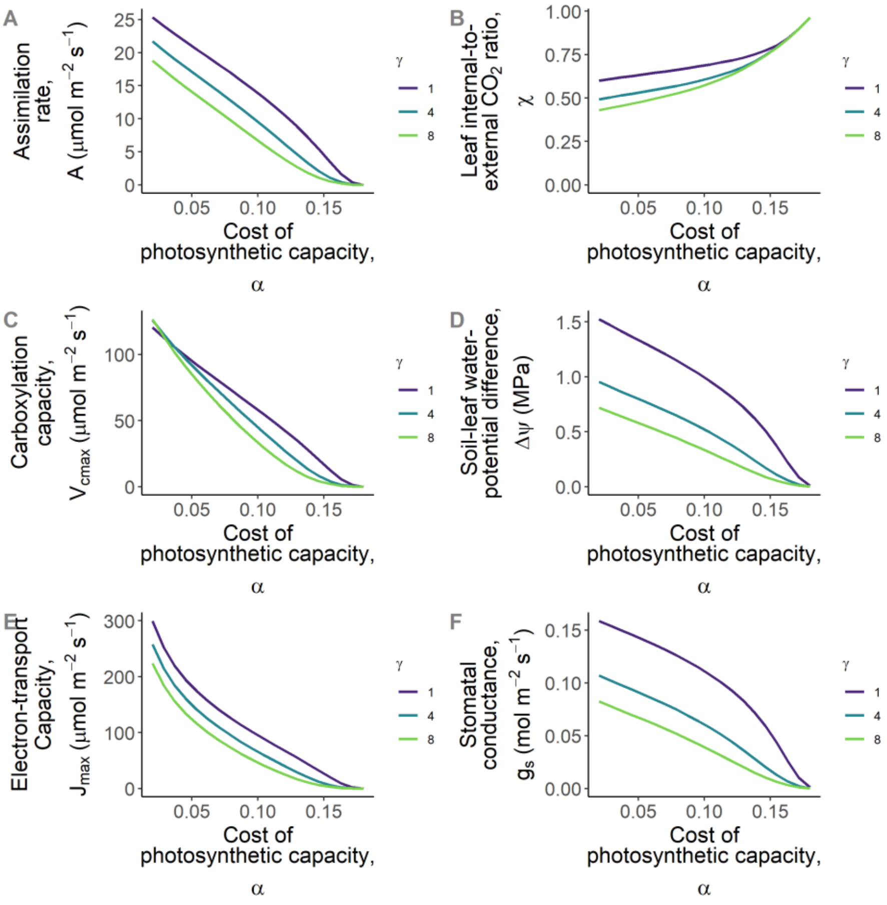

Fig. S5. Sensitivity of model predictions to the cost parameters. Predicted model variables as a function of the photosynthetic costs α and hydraulic costs γ: (A) assimilation rate A, (B) leaf-internal-to-external CO_2 ratio χ, (C) carboxylation capacity V_{cmax}, (D) soil-to-leaf water potential difference $\Delta\psi$, (E) electron transport capacity J_{max}, and (F) stomatal conductance g_s. All variables vary strongly with the cost of photosynthetic capacity (α) whereas only g_s and $\Delta\psi$ additionally vary with the hydraulic costs (γ). The following parameters were used in this plot: $T = 25^o C$, $I_{\mathrm{abs}} = 210$ $\mu mol\ m^{-2}\ s^{-1}$, VPD = 1 kPa, CO_2 = 400 ppm, $\psi_s = 0$, $b_r = 0$, $K = 0.3 \times 10^{-16}\ m^2$, $\psi_{50} = -2$ MPa, $b = 2$.

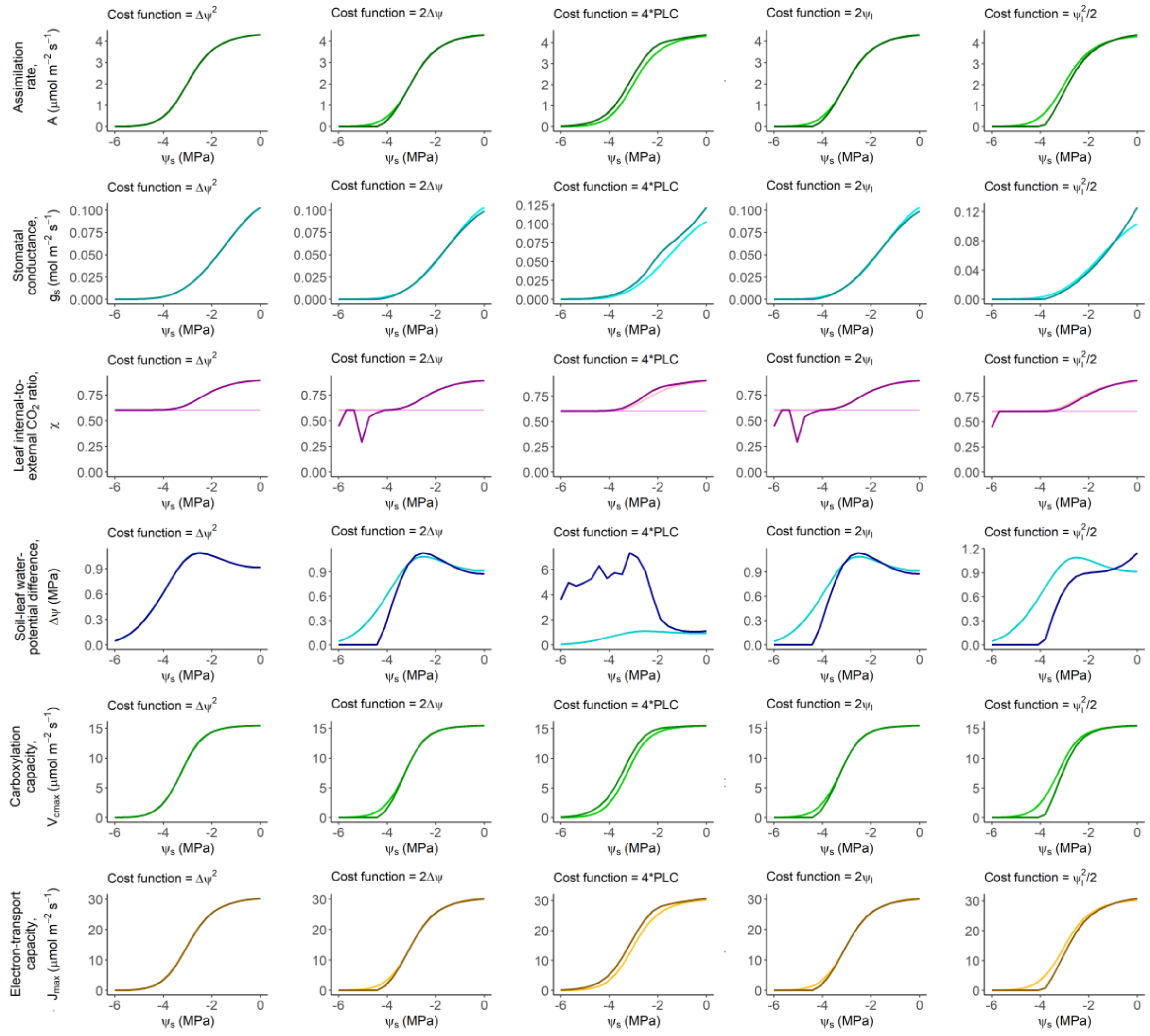

Fig. S6. Sensitivity of model predictions to the form of the hydraulic cost function. Predicted model variables (in rows) as a function of soil water potential ψ_s for different formulations of the hydraulic cost function (in columns). Light coloured lines represent the original model with a cost function of $\Delta\psi^2$, whereas dark coloured lines represent predictions with the modified cost function as specified in panel titles. Overall, predictions of A, V_{cmax}, J_{max}, χ, and g_s are robust to the choice of the cost function, whereas predictions of $\Delta\psi$ show greater variability, and thus form the basis for choosing the appropriate function. Cost functions $\Delta\psi$ and $\Delta\psi^2$ give similar results and can both be good candidates. However, we have chosen $\Delta\psi^2$ over $\Delta\psi$ because the latter predicts a sharp stomatal closure point beyond which both A and g_s become zero, whereas observations show a smoother decline. The cost functions ψ_l^2 and PLC ($= 1 - P(\psi)$), have been used in many stomatal models, but give unrealistic predictions of $\Delta\psi$ in our model. Other parameters used for this plot are the same as in Fig. S5.

4 Cross-validation

Species	E_r (train) (mean ± st. dev.)	E_r (test) (mean ± st. dev.)	E_r (train) (median)	E_r (test) (median)	Number of data points
Allocasuarina luehmannii	19.41 ± 2.78	24.94 ± 17.26	19.69	18.27	23
Eucalyptus pilularis	30.61 ± 5.05	41.18 ± 16.88	28.19	44.66	54
Eucalyptus populnea	14.42 ± 0.49	15.53 ± 1.89	14.20	15.92	65
Glycine max	41.58 ± 10.56	118.90 ± 154.64	41.69	48.61	9
Pseudotzuga menziesii	21.19 ± 5.27	87.62 ± 100.83	23.43	22.80	5
Quercus coccifera	59.32 ± 15.11	309.20 ± 347.01	53.45	134.94	6
Quercus ilex	38.10 ± 2.88	56.84 ± 16.88	39.31	49.20	5
Quercus suber	17.81 ± 1.86	72.00 ± 111.06	18.53	20.43	5

Table S1. Cross-validation results for species with complete data. We have tested the performance of our model using five-fold cross-validation. For each species for which data was available on the soil-water potential difference $\Delta\psi$, we divided the data on the assimilation rate A, the stomatal conductance g_s, and the leaf internal-to-external CO_2 ratio χ into five sets. We then performed five training iterations, such that in each iteration we used one of the five sets for testing and the remaining four for training, i.e., estimating parameters. All data on $\Delta\psi$ was used for error calculations. For species with around five data points, each set has only one data point, which makes the algorithm equivalent to leave-one-out cross-validation. The error E_r is calculated according to Eq. 8. Median errors are comparable across the training and testing datasets, suggesting that our model generalizes well to out-of-sample environmental conditions.

5 Sources of empirical trait values

**Species**	$\tilde{SLA}$	ψ_{50x}
Allocasuarina luehmannii	TRY	
Helianthus annuus	TRY	MS
Cedrus atlantica	TRY	MS
Pseudotzuga menziesii	BN	MS
Glycine max	TRY	
Olea europaea var. Meski	TRY	MA
Olea europaea var. Chemlali	TRY	MS
Quercus coccifera	TRY	MA
Quercus suber	TRY	MS
Broussonetia papyrifera	L11	TRY
Eucalyptus pilularis	AC	
Eucalyptus populnea	TRY	
Ficus tikoua		MS
Cinnamomum bodinieri	L11	
Platycarya longipes	L11	BA
Pteroceltis tatarinowii	L11	BA
Rosa cymosa	L11	
Quercus ilex	TRY	MS

BN - [83] AC - [84] MS - [40] TRY - [85] BA - [25] MA - [86] L11 - [87]

Table S2. Sources of the empirical values of the specific leaf area $S\tilde{L}A$ and the water potential ψ_{50x} at which 50% conductivity is lost in the xylem (Fig. 1).

6 References

81. Choat, B., Ball, M. C., Luly, J. G. & Holtum, J. A. M. Hydraulic architecture of deciduous and evergreen dry rainforest tree species from north-eastern Australia. *Trees* **19**, 305–311 (2005).
82. West, G. B., Brown, J. H. & Enquist, B. J. A general model for the structure and allometry of plant vascular systems. *Nature* **400**, 4 (1999).
83. Bansal, S., Harrington, C. A., Gould, P. J. & St.Clair, J. B. Climate-related genetic variation in drought-resistance of Douglas-fir (Pseudotsuga menziesii). *Global Change Biology* **21**, 947–958 (2015).
84. Alcorn, P. J. *et al.* Crown structure and vertical foliage distribution in 4-year-old plantation-grown Eucalyptus pilularis and Eucalyptus cloeziana. *Trees* **27**, 555–566 (2013).
85. Kattge, J. *et al.* TRY – a global database of plant traits. *Global Change Biology* **17**, 2905–2935 (2011).
86. Manzoni, S., Vico, G., Katul, G., Palmroth, S. & Porporato, A. Optimal plant water-use strategies under stochastic rainfall. *Water Resources Research* **50**, 5379–5394 (2014).

87. Liu, C.-C. *et al.* Comparative ecophysiological responses to drought of two shrub and four tree species from karst habitats of southwestern China. *Trees* **25**, 537–549 (2011).
