# Supplementary material for: Towards a unified theory of plant photosynthesis and hydraulics
Source: Nat Plants. 2022 Oct 27;8(11):1304–16. doi: 10.1038/s41477-022-01244-5 (PMC9663302; doi:10.1038/s41477-022-01244-5)
Supplement: Supplementary file 2 — Reporting Summary. [file 41477_2022_1244_MOESM2_ESM.pdf]

nature portfolio

Corresponding author(s): Jaideep Joshi

Last updated by author(s): Jul 8, 2022

# Reporting Summary

Nature Portfolio wishes to improve the reproducibility of the work that we publish. This form provides structure for consistency and transparency in reporting. For further information on Nature Portfolio policies, see our Editorial Policies and the Editorial Policy Checklist.

## Statistics

For all statistical analyses, confirm that the following items are present in the figure legend, table legend, main text, or Methods section.

| n/a | Confirmed | |
|---|---|---|
| ☒ | ☐ | The exact sample size (*n*) for each experimental group/condition, given as a discrete number and unit of measurement |
| ☒ | ☐ | A statement on whether measurements were taken from distinct samples or whether the same sample was measured repeatedly |
| ☒ | ☐ | The statistical test(s) used AND whether they are one- or two-sided<br>*Only common tests should be described solely by name; describe more complex techniques in the Methods section.* |
| ☒ | ☐ | A description of all covariates tested |
| ☐ | ☒ | A description of any assumptions or corrections, such as tests of normality and adjustment for multiple comparisons |
| ☐ | ☒ | A full description of the statistical parameters including central tendency (e.g. means) or other basic estimates (e.g. regression coefficient) AND variation (e.g. standard deviation) or associated estimates of uncertainty (e.g. confidence intervals) |
| ☒ | ☐ | For null hypothesis testing, the test statistic (e.g. *F*, *t*, *r*) with confidence intervals, effect sizes, degrees of freedom and *P* value noted<br>*Give P values as exact values whenever suitable.* |
| ☒ | ☐ | For Bayesian analysis, information on the choice of priors and Markov chain Monte Carlo settings |
| ☒ | ☐ | For hierarchical and complex designs, identification of the appropriate level for tests and full reporting of outcomes |
| ☒ | ☐ | Estimates of effect sizes (e.g. Cohen's *d*, Pearson's *r*), indicating how they were calculated |

*Our web collection on statistics for biologists contains articles on many of the points above.*

## Software and code

Policy information about availability of computer code

| | |
|---|---|
| Data collection | No new data collection was done in this manuscript. Online figure digitizer (https://automeris.io/WebPlotDigitizer/) was used to extract data from published sources. |
| Data analysis | Custom code used for the analysis is available on GitHub: https://github.com/jaideep777/phydro |

For manuscripts utilizing custom algorithms or software that are central to the research but not yet described in published literature, software must be made available to editors and reviewers. We strongly encourage code deposition in a community repository (e.g. GitHub). See the Nature Portfolio guidelines for submitting code & software for further information.

## Data

Policy information about availability of data

All manuscripts must include a data availability statement. This statement should provide the following information, where applicable:

- Accession codes, unique identifiers, or web links for publicly available datasets
- A description of any restrictions on data availability
- For clinical datasets or third party data, please ensure that the statement adheres to our policy

The following data-availability statement is included in the manuscript: All data used in this manuscript are compiled from the literature. We have provided citations to publications and databases at appropriate locations in the manuscript. The compiled database can be found in the supplementary information.

# Field-specific reporting

Please select the one below that is the best fit for your research. If you are not sure, read the appropriate sections before making your selection.

☐ Life sciences ☐ Behavioural & social sciences ☒ Ecological, evolutionary & environmental sciences

For a reference copy of the document with all sections, see nature.com/documents/nr-reporting-summary-flat.pdf

# Ecological, evolutionary & environmental sciences study design

All studies must disclose on these points even when the disclosure is negative.

| | |
|---|---|
| Study description | In this study, we develop a new optimality-based theory for plant photosynthesis and hydraulics. |
| Research sample | Data extracted from published literature. A table of data sources is included in the manuscript. |
| Sampling strategy | Sampling strategy does not apply as we used data from the literature. |
| Data collection | Literature was searched for triplets of assimilation rate, stomatal conductance, and predawn leaf water potential in drydown experiments. Data was extracted using online digitization tools. |
| Timing and spatial scale | Not applicable as published data on experiments was used. |
| Data exclusions | No data were excluded. |
| Reproducibility | Code to reproduce our key results is available in the vignettes folder of our repo: https://github.com/jaideep777/phydro/tree/master/vignettes |
| Randomization | Not applicable as repeated measurements on the same plants were desired and control experiments were not necessary. |
| Blinding | Not applicable as treatment vs control paradigm is not applicable. |

Did the study involve field work? ☐ Yes ☒ No

# Reporting for specific materials, systems and methods

We require information from authors about some types of materials, experimental systems and methods used in many studies. Here, indicate whether each material, system or method listed is relevant to your study. If you are not sure if a list item applies to your research, read the appropriate section before selecting a response.

## Materials & experimental systems

| n/a | Involved in the study | |
|---|---|---|
| ☒ | ☐ | Antibodies |
| ☒ | ☐ | Eukaryotic cell lines |
| ☒ | ☐ | Palaeontology and archaeology |
| ☒ | ☐ | Animals and other organisms |
| ☒ | ☐ | Human research participants |
| ☒ | ☐ | Clinical data |
| ☒ | ☐ | Dual use research of concern |

## Methods

| n/a | Involved in the study | |
|---|---|---|
| ☒ | ☐ | ChIP-seq |
| ☒ | ☐ | Flow cytometry |
| ☒ | ☐ | MRI-based neuroimaging |
